# Evaluation of Expression and Clinicopathological Relevance of Small Nucleolar RNAs (snoRNAs) in Invasive Breast Cancer †

**DOI:** 10.3390/ncrna11060076

**Published:** 2025-10-31

**Authors:** Luděk Záveský, Eva Jandáková, Vít Weinberger, Luboš Minář, Radovan Turyna, Adéla Tefr Faridová, Veronika Hanzíková, Ondřej Slanař

**Affiliations:** 1 Institute of Biology and Medical Genetics, First Faculty of Medicine, Charles University, Prague and General University Hospital in Prague, Albertov 4, 128 00 Prague, Czech Republic; 2 Institute of Pharmacology, First Faculty of Medicine, Charles University Prague, General University Hospital in Prague, Albertov 4, 128 00 Prague, Czech Republic; ondrej.slanar@vfn.cz; 3 Department of Pathology, Faculty of Medicine, Masaryk University and University Hospital Brno, Obilní trh 11, 602 00 Brno, Czech Republic; jandakova.eva@fnbrno.cz; 4 Department of Obstetrics and Gynecology, Masaryk University, University Hospital Brno, Obilní trh 11, 602 00 Brno, Czech Republic; weinberger.vit@fnbrno.cz (V.W.); minar.lubos@fnbrno.cz (L.M.); 5 Institute for the Care of Mother and Child, Podolské nábřeží 157/36, 147 00 Prague, Czech Republic; radovan.turyna@lf3.cuni.cz (R.T.); adela.faridova@gmail.com (A.T.F.); 6 Third Faculty of Medicine, Charles University, 100 00 Prague, Czech Republic; 7 Institute for Postgraduate Medical Education—IPVZ, 100 00 Prague, Czech Republic; 8 Faculty Transfusion Department, General University Hospital in Prague, 128 08 Prague, Czech Republic; veronika.hanzikova@vfn.cz

**Keywords:** biomarker, breast cancer, small nucleolar RNA, SCARNA2, SCARNA3, SNORD15B, SNORD94, SNORA68, SNHG1, RNU2-1

## Abstract

**Background/Objectives:** Breast cancer is a leading cause of cancer-related mortality among women worldwide. Small nucleolar RNAs (snoRNAs) represent a class of non-coding RNAs with potential as novel biomarkers applicable to improve diagnostic and prognostic applications. **Methods:** We performed a comprehensive evaluation of the snoRNA-related gene expression by qPCR using benign and tumor tissue samples associated with invasive breast carcinomas of no special type (NST). Selected candidate snoRNAs, i.e., SCARNA2, SCARNA3, SNORD15B, SNORD94, SNORA68, and SNHG1, along with RNU2-1 snRNA, were further validated and their associations with clinicopathological parameters were examined. External datasets and plasma samples were used for additional validation. **Results:** SCARNA2 was identified as the most promising snoRNA biomarker candidate, showing a positive association with better progression-free survival (PFS) in our data (13.3-month survival difference between low- and high-expression groups) and with both PFS and overall survival in external RNA-seq datasets. SNORD94, SNORD15B, SCARNA3, and RNU2-1 snRNA were also indicated as putative tumor suppressors. SNORD94 was associated with better progression-free survival (PFS) in our data as well (12.4-month survival difference between low- and high expression groups). Greater downregulation in the low-expression tumor subgroup compared to benign samples further supports the prognostic potential of SCARNA2 and SNORD94. Evidence for SNHG1 and SNORA68 as putative oncogenes was less conclusive. **Conclusions:** Several small nucleolar RNAs were found to be dysregulated in breast cancer specimens, supporting their further evaluation as potential biomarkers. In particular, SCARNA2, SNORD94, SNORD15B, SCARNA3, and RNU2-1 snRNA merit further investigation to determine their clinical relevance and biological roles in breast cancer.

## 1. Introduction

Breast cancer is the most commonly diagnosed malignancy in women. In 2022, it accounted for approximately 2.3 million new cases globally and remained the leading cause of cancer-related mortality among women, with nearly 666,000 deaths reported in the same year. Moreover, recent epidemiological data show a continuous increase in breast cancer incidence [1,2]. Addressing this burden requires the development of new diagnostic, predictive, prognostic, and therapeutic options.

Breast cancer is a complex disease encompassing two major categories: invasive ductal carcinoma of no special type (NST breast carcinomas) and lobular carcinoma. NST breast carcinomas, which account for the majority of cases, are further classified into four major subtypes (Luminal A, Luminal B, HER2-enriched, triple-negative/basal-like) based on histopathological features, including the expression of estrogen and progesterone receptors, HER2 status, and the Ki-67 proliferation marker. Similar subtypes are also recognized using the PAM50 molecular classification based on the gene signature of 50 genes [3]. However, the molecular heterogeneity of breast cancer is likely more complex [4], highlighting the need for further research to identify gene signatures across diverse groups of genes, including both protein-coding and non-coding genes. The latter category involves various classes of regulatory RNA molecules such as small non-coding RNAs and long non-coding RNAs. In recent years, many differentially expressed genes have been proposed as novel candidate biomarkers with potential diagnostic and prognostic applications in breast cancer. Our recent studies, for instance, identified such biomarkers in plasma-derived microRNAs [5], as well as in tumor tissues among human endogenous retroviruses [6], long non-coding RNAs [7], microRNAs [8] and genes related to miRNAs and apoptosis [9]. In the course of these studies, we identified a gap in knowledge about small nucleolar RNAs in breast cancer.

In other classes of non-coding RNAs, candidate biomarkers have already been identified in various sample types, such as tissues or liquid biopsies, often showing promising sensitivity, specificity, and associations with patient survival [10]. However, knowledge regarding snoRNAs remains limited. Small nucleolar RNAs (snoRNAs) thus represent a relatively understudied class of small non-coding RNAs, approximately 60–300 nucleotides in length. They are classified into two major families, namely C/D box snoRNAs (SNORDs) and H/ACA box snoRNAs, along with a subset known as small Cajal body-specific RNAs (scaRNAs), which include members of both aforementioned groups and are localized in the nucleoplasmic Cajal bodies. Moreover, some snoRNAs are encoded within introns of specific long-non-coding RNAs (small nucleolar RNA host genes, SNHGs) [11].

SnoRNAs have diverse biological functions, primarily guiding chemical modifications of other RNAs, including ribosomal RNAs, transfer RNAs, and small nuclear RNAs, while also participating in ribosome biogenesis and post-transcriptional RNA modifications. Research has indicated that snoRNAs may also be involved in the regulation of chromatin structure and other biological processes [12,13].

While accumulating evidence suggests the involvement of particular snoRNAs and related genes in breast carcinogenesis, our understanding of their precise biological roles and actual clinical impact remains largely limited. Nevertheless, several studies have also revealed their diagnostic and prognostic potential in breast cancer, suggesting that their further exploration may be valuable for the discovery of novel biomarkers based on the related RNA molecules [14,15].

In this study, we performed a large-scale expression screening of 42 selected snoRNA-related RNAs representing seven SNORD RNAs (small nucleolar RNA, C/D Box, snoRNA), two H/ACA box snoRNAs, 21 scaRNAs (small Cajal body-specific RNAs), 10 small nucleolar RNA host gene RNAs (SNHGs, i.e., long non-coding RNAs), and additionally, one snRNA (RNU2-1) and TERC (telomerase RNA, also representing small Cajal body-specific RNAs). Their expression was assessed in tumor tissues of invasive breast cancer of no special type (NST) and compared with benign samples. Six candidate snoRNA-related genes (SCARNA2, SCARNA3, SNORD15B, SNORD94, SNORA68, and SNHG1) and RNU2-1 snRNA were validated, and associations with clinicopathological data were investigated. The results were further validated using external datasets. Based on these findings, the expression of five genes was also evaluated in blood plasma samples to explore their diagnostic potential. We identified several candidate non-coding RNA genes that warrant further investigation as potential biomarkers in breast cancer.

## 2. Results

### 2.1. Tissue Experiment

#### 2.1.1. Differentially Expressed Genes in the Screening

In the screening phase, a selected part of all samples available was analyzed. Eight tumor samples were compared with their eight benign counterparts using custom-designed qPCR arrays, each covering two samples per array for a selection of snoRNA-related genes. In total, 42 tested genes including RNU2-1 (small nuclear RNA, interacting with the 3′ region of the intron at the branch site, necessary for splicing) and TERC (SCARNA/telomerase RNA class, important in maintaining the length of telomeres), were analyzed.

In the screening, a number of candidate genes were found to be differentially expressed between tumor and benign samples across the two Ct cut-offs and two normalization procedures. Most candidate genes appeared downregulated in tumor samples. Among these candidate tumor suppressors, SNORD94, RNU2-1, SCARNA3, SNORD15B, and SCARNA2 appeared among the most promising for further validation, as their dysregulation was high and provided consistent results. Similarly, SNORA68 and SNHG1 were selected as candidate oncogenes. Within the screening results, other gene candidates for follow-up studies can be found as well. To note several examples, SCARNA17, SNORD48, SNORD97, and SNORD89 appeared as promising downregulated genes comparing tumor and benign breast samples, while SCARNA22 and SCARNA27 may be promising upregulated genes. See Supplementary File S1 (Table S1) for details.

#### 2.1.2. Differentially Expressed Genes in the Validation

**Figure 1:**
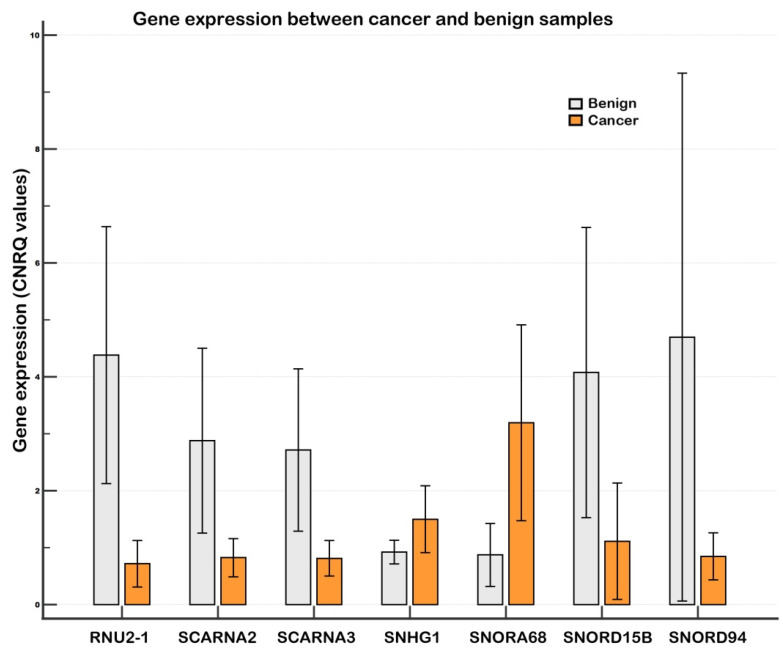
Relative gene expression between tumor and benign breast tissue samples (Experiment Tissue, validation). Notes: A clustered multiple-comparison graph showing gene expression data (non-log-transformed CNRQs). Error bars indicate 95% CI for the mean. CNRQs = calibrated normalized relative quantities calculated in qbase+. Data were normalized with the reference genes (*GAPDH*, *GUSB*, and *HPRT1*). Tumor samples: *n* = 22, benign samples: *n* = 20.

**Figure 2:**
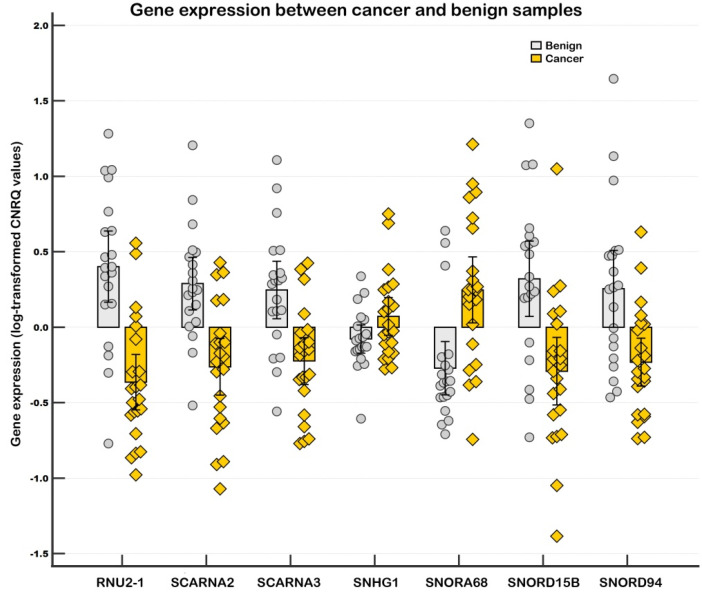
Relative gene expression between tumor and benign breast tissue samples in individual samples (Experiment Tissue, validation). Notes: A clustered multiple-comparison graph showing gene expression data (log-transformed CNRQs). Error bars indicate 95% CI for the mean. CNRQs = calibrated normalized relative quantities calculated in qbase+. Data were normalized with the reference genes (*GAPDH*, *GUSB*, and *HPRT1*). Tumor samples: *n* = 22, benign samples: *n* = 20.

In the validation phase, we used an extended set of samples (tumor samples, *n* = 22; benign samples, *n* = 20). All investigated genes were found to be significantly altered congruently with the screening results, except for SNHG1, which showed significant differences only when global mean normalization was applied. The strongest downregulation between tumor and benign samples in the validation phase was observed for RNU2-1 snRNA (–5.8-fold with endogenous control normalization, –4.5-fold with global mean normalization). Among snoRNAs, SNORD15B was the most strongly downregulated, while SNORA68 showed the highest upregulation (i.e., 3.3-fold with endogenous control normalization and 4.3-fold with global mean normalization). See Table 1 and Figure 1 and Figure 2 for details.

#### 2.1.3. Diagnostic Performance of the Investigated Genes

Receiver Operating Characteristic (ROC) analysis was performed to calculate the area under the curve (AUC), sensitivity, and specificity in order to evaluate the diagnostic potential of the investigated genes.

The best diagnostic performance was observed for RNU2-1 (AUC = 0.873; sensitivity: 90.91%, specificity: 80%). Strong performance was also found for SCARNA2 (AUC = 0.845; sensitivity: 72.73%, specificity: 90%), SCARNA3 (AUC = 0.805; sensitivity: 86.36%, specificity: 75%), SNORA68 (AUC = 0.805; sensitivity: 77.27%, specificity: 85%), and SNORD15B (AUC = 0.807; sensitivity: 86.36%, specificity: 75%).

Two genes, SNHG1 and SNORD94, showed suboptimal diagnostic performance. SNHG1 had a lower AUC (0.632) and poor sensitivity (45.45%) despite relatively good specificity (85%). SNORD94 reached an AUC of 0.770 and high sensitivity (86.36%), but its specificity was limited (60%). See Supplementary File S2 (Table S2) for details.

#### 2.1.4. Associations with the Clinicopathological Characteristics

##### Stage and Grade

Regarding tumor stage, SCARNA2 was significantly upregulated between stage I (*n* = 13) and stage IV (*n* = 2) (*p* = 0.0272), and showed a similar trend between stage I (*n* = 13) and II (*n* = 7) (*p* = 0.0522) and stage II (*n* = 7) vs. IV (*n* = 2) (*p* = 0.0790). SNORD15B was significantly upregulated between stage I and IV (*p* = 0.0272), and marginally significantly upregulated between stage II and IV (*p* = 0.0790). RNU2-1 also showed significant upregulation between stage I and IV (*p* = 0.0272) and stage II and IV (*p* = 0.0404). SCARNA3 showed marginally significant upregulation when comparing stage I vs. II (*p* = 0.1223) and stage I vs. IV (*p* = 0.0894). SNORA68 showed a trend to downregulation between stage I and IV (*p* = 0.1264) and between stage II and IV (*p* = 0.0790).

**Figure 3:**
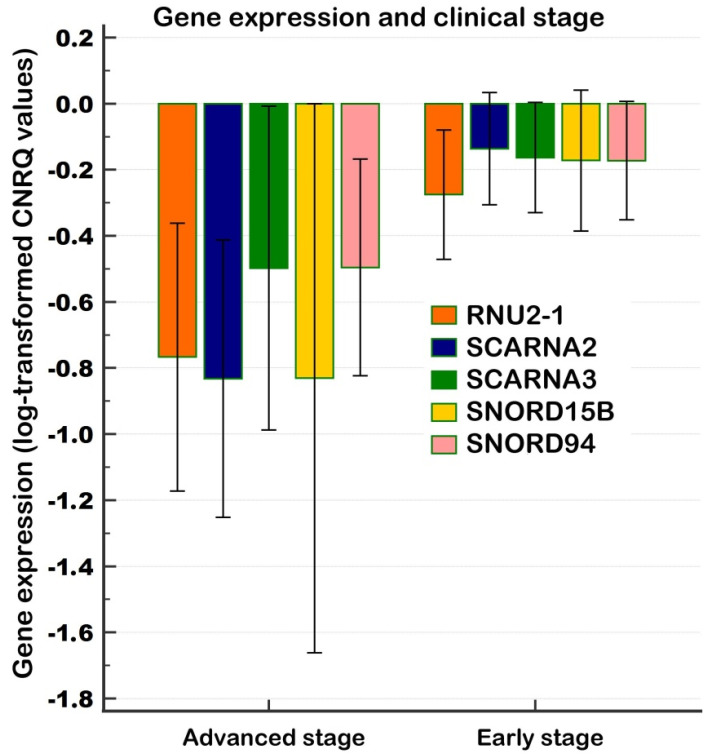
Relative gene expression between tumor samples of early and advanced clinical stages (Experiment Tissue, validation). Notes: A clustered multiple-variables graph showing gene expression data (log-transformed CNRQs) for five representative genes. Error bars indicate 95% CI for the mean. CNRQs = calibrated normalized relative quantities calculated in qbase+. Data were normalized with the reference genes (*GAPDH*, *GUSB*, and *HPRT1*). Early-clinical-stage samples (*n* = 18); advanced-clinical-stage samples (*n* = 4).

Due to limited sample numbers in aforementioned analyses, we next compared samples based on clinical stage, categorized as early (*n* = 18) and advanced (*n* = 4). Significantly higher expressions of RNU2-1 (*p* = 0.0269), SCARNA2 (*p* = 0.0064), and SNORD15B (*p* = 0.0411), along with a trend to higher levels of SCARNA3 (p = 0.0738) and SNORD94 (*p* = 0.0611), were observed in early-stage samples. The results are depicted in Figure 3 and should be considered as tentative due to limited number of advanced-stage samples.

**Figure 4:**
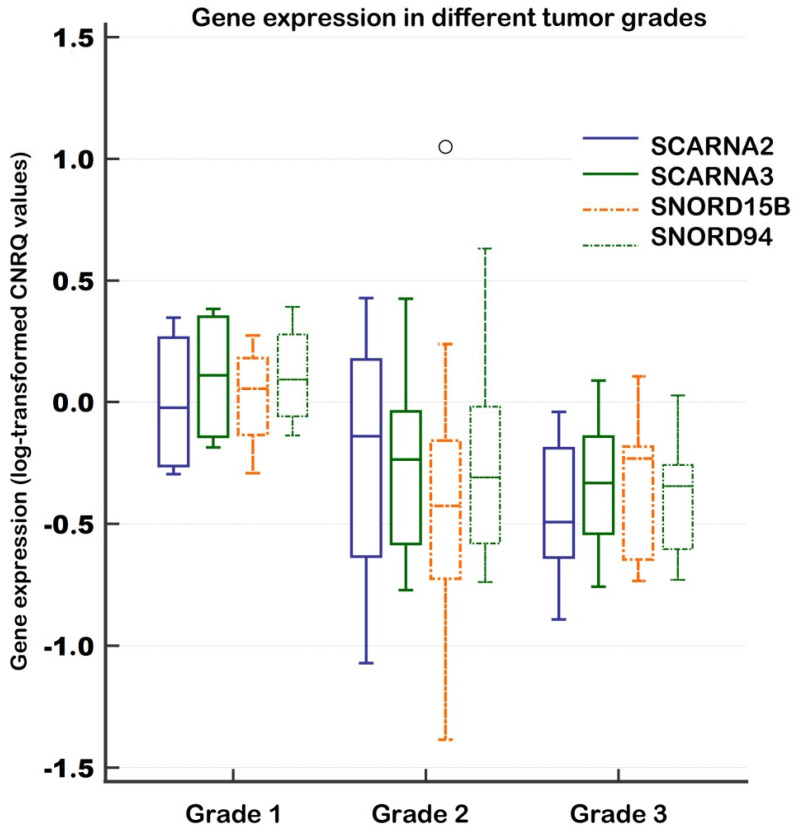
Relative gene expression between tumor samples of different grades (Tissue experiment, validation). Notes: A clustered multiple-variables graph showing gene expression data (log-transformed CNRQs) for four representative genes. A box of the box-plot is drawn from the 1st to 3rd quartile (the 25th and 75th percentiles). A horizontal line within a box plot represents the median. Horizontal lines are drawn at the highest value and the lowest expression value. CNRQs = calibrated normalized relative quantities calculated in qbase+. Data were normalized with the reference genes (*GAPDH*, *GUSB*, and *HPRT1*). Tumor grade 1 (*n* = 4); tumor grade 2 (*n* = 10); tumor grade 3 (*n* = 8).

Comparing different tumor grades, significantly higher expression in grade 1 (*n* = 4) compared to grade 3 (*n* = 8) was observed for SCARNA3 (*p* = 0.0415) and SNORD94 (*p* = 0.0174). Trends with marginally significant differences between grade 1 (higher expression) and grade 2 (*n* = 10) (lower expression) were noted for SCARNA3 (*p* = 0.1573), SNORD15B (*p* = 0.0897), and SNORD94 (*p* = 0.0660). In comparisons between grade 1 and grade 3, lower expression levels in grade 3 were also observed for SCARNA2 (*p* = 0.0617) and SNORD15B (*p* = 0.1264). The results for these genes are depicted in Figure 4.

##### Multifocal Disease and Lymph Node Metastases (LNMs)

SNORD15B showed lower levels in multifocality-positive samples (*n* = 3) compared with negative samples (*n* = 19) approaching statistical significance (*p* = 0.0941). Significantly lower expression of RNU2-1 was observed in lymph node metastasis-positive samples (*n* = 4) compared with negative samples (*n* = 14) (*p* = 0.0337), along with a trend toward lower levels of SCARNA2 (*p* = 0.0710) and SNORD15B (*p* = 0.0559), compared to LNM-negative samples. A similar trend was noted for SNORD94 (*p* = 0.1922).

##### Estrogen (ER) and Progesterone (PR) Receptors, Ki-67 Proliferation Marker

Concerning hormone receptors, we observed a negative association between SNHG1 expression and ER positivity (*n* = 20 positive samples vs. two negative samples) at both 1% and 10% cut-offs; *p* = 0.0398), PR positivity (*n* = 6 negative samples, *n* = 16 positive samples using 10% cut-off; *p* = 0.0328), and combined ER+/PR+ status (1% cut-off; *n* = 2 negative, *n* = 17 positive, *p* = 0.0468; 10% cut-off; *n* = 2 negative, *n* = 16 positive, *p* = 0.0246). See Supplementary File S3 for the corresponding figures.

No statistically significant associations were observed for Ki-67 status. When a 15% cut-off was applied, 11 positive and 11 negative samples were compared; for a 20% cut-off, the comparison included 10 positive and 12 negative samples. SCARNA2 showed a suggestive negative association with Ki-67 positivity at both the 15% (*p* = 0.0818) and 20% (*p* = 0.0750) cut-offs. Similarly, SNORD94 expression was lower in Ki-67-positive samples (15% cut-off; *p* = 0.0613), and SCARNA3 was downregulated in Ki-67-positive samples at the 20% cut-off (*p* = 0.1469).

When comparing luminal A and luminal B samples, we observed significantly reduced expression of SNORD94 in luminal B samples (*p* = 0.0367). A trend toward downregulation was also observed for all other candidate tumor suppressors: RNU2-1 (*p* = 0.1599), SCARNA2 (*p* = 0.0627), SCARNA3 (*p* = 0.1194), and SNORD15B (*p* = 0.0742).

As the above-mentioned analyses were based on relatively limited sample numbers in comparisons, we further evaluated these associations using RNAseq data. It is noteworthy that the negative association of SNHG1 and ER/PR positivity was confirmed in RNAseq data (see later).

#### 2.1.5. Gene Expression in Tissues and Survival

The impact of investigated gene expression on overall survival (OS) and progression-free survival (PFS) was assessed using Kaplan–Meier analyses and 21 samples with available survival data (clinical early stage, *n* = 18; clinical advanced stage, *n* = 3). Although no statistically significant associations were observed for OS, a trend toward improved survival in the high expression group was noted for RNU2-1, SCARNA2, SCARNA3, and SNORD94, whereas SNORA68 showed the opposite trend, with worse OS in the high-expression group.

Two significant associations were observed for PFS, both indicating a positive impact of higher gene expression on survival outcomes. For SCARNA2 (*p* = 0.0475), the mean PFS was 272.1 weeks (62.6 months) in the low-expression group versus 330.0 weeks (75.9 months) in the high-expression group (i.e., 13.3-month difference), with all five progression events recorded in the low expression group. Similarly, SNORD94 (*p* = 0.0475) showed a mean PFS of 272.1 weeks (62.6 months) in the low-expression group versus 326.0 weeks (75 months) in the high-expression group (i.e., 12.4-month difference), with all five events again occurring in the low-expression group.

**Figure 5:**
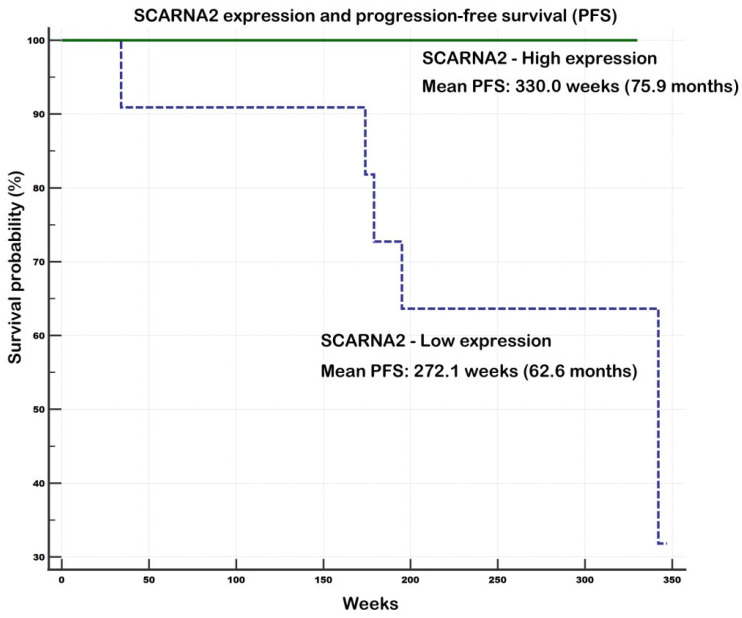
Progression-free survival in relation to *SCARNA2* expression levels. Notes: Univariate Kaplan–Meier survival curves for progression-free survival (PFS) related to low and high concentrations of *SCARNA2* in invasive breast carcinoma NST tumors. All five survival endpoints were noted in low expression group of patients. *SCARNA2* appears as a favorable prognostic factor for PFS. Survival data included clinical early stage (*n* = 18); clinical advanced stage (*n* = 3).

**Figure 6:**
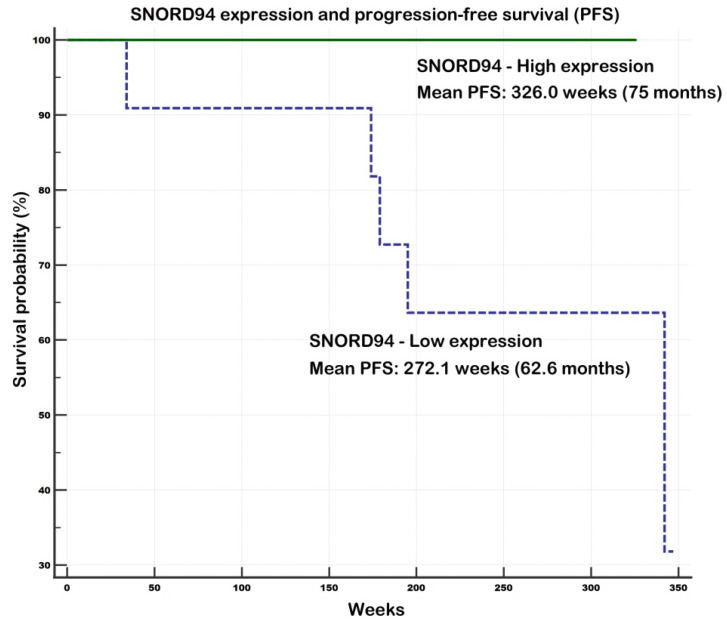
Progression-free survival in relation to *SNORD94* expression levels. Notes: Univariate Kaplan–Meier survival curves for progression-free survival (PFS) related to low and high concentrations of *SNORD94* in invasive breast carcinoma NST tumors. All five survival endpoints were noted in low expression group of patients. *SNORD94* appears as a favorable prognostic factor for PFS. Survival data included clinical early stage (*n* = 18); clinical advanced stage (*n* = 3).

Additionally, four out of five progression events were recorded in the low-expression group for RNU2-1 and SCARNA3, indicating a similar trend despite the lack of statistical significance. See Supplementary File S4 (Table S3) and Figure 5 (SCARNA2—PFS) and Figure 6 (SNORD94—PFS) for details.

##### Evaluation of Prognostic Potential of SCARNA2 and SNORD94

As indicated above, SCARNA2 and SNORD94 may possess prognostic potential based on tumor samples. Therefore, we next tested whether the low-expression and high-expression samples provide different ratios also between cancer samples and benign samples.

Indeed, we found that low-expression samples exhibited markedly greater downregulation in both genes when comparing cancer and benign samples, whereas high-expression samples showed no significant differences compared to benign samples. In the low-expression cancer group (*n* = 11) compared to benign samples (*n* = 20), SCARNA2 showed a fold difference (FD) of −7.3 (*p* = 2.83 × 10^−7^), and SNORD94 had an FD of −5.75 (*p* = 0.00001). In contrast, in the high-expression cancer group (*n* = 10) versus benign samples (*n* = 20), SCARNA2 exhibited an FD of −1.55 (*p* = 0.109), and SNORD94 had an FD of −1.51 (*p* = 0.502).

### 2.2. External Validation

#### 2.2.1. Differential Gene Expression Across Different Subtypes

The expression patterns of the validated snoRNA-associated genes and RNU2-1 snRNA were further evaluated using Breast Cancer Gene-Expression Miner v5.2 (bc-GenExMiner v5.2) [16,17,18], which analyzes RNAseq and DNA microarray datasets for transcriptomic data from breast cancer patients and their tissue samples.

Using TCGA + GTEx data for the available genes, lower expression levels of SCARNA3, SNORD94, SNHG1, and SNORD15B were observed in tumor samples compared with healthy controls. However, no significant differences were found between tumors and adjacent tissues. Interestingly, SNHG1 showed an opposite trend toward upregulation in our data (significant when global mean normalization was applied). Other discrepancies may result from variations in the compared sample sets, as the GTEx cohort represents an independent population and not benign tissues from breast cancer patients. In our study, most samples corresponded to early-stage luminal A and luminal B tumors, which may also explain the absence of significant differences observed when analyzing the overall datasets. Indeed, RNAseq data (TCGA/ScanB datasets) analyzing PAM50 breast cancer subtypes revealed variable expression patterns. For example, SCARNA2 showed higher expression in luminal A samples compared with HER2-enriched and basal-like subtypes. SNORD15B had lower expression in luminal A versus basal-like samples. SNHG1 showed lower expression in luminal A compared with basal-like and HER2-enriched samples, and also in luminal B compared with basal-like samples. Interestingly, SNHG1 was also reported to be lower in luminal A compared with luminal B samples.

Using DNA microarray transcriptomic data, several significant findings were observed. SCARNA2 expression was lower in luminal A samples compared with both basal-like and normal breast-like samples. SCARNA3 showed higher expression in luminal A compared with HER2-enriched and luminal B subtypes, and differed also between basal-like and HER2-enriched samples. SNORD94 levels were higher in normal breast-like and luminal A samples compared with basal-like, and also higher in normal breast-like compared to HER2-enriched samples. SNORD15B exhibited lower expression in luminal A compared with basal-like, HER2-enriched, and luminal B subtypes. SNORA68 expression was elevated in luminal A, luminal B, and normal breast-like samples compared with HER2-enriched. Higher levels of SNORA68 were noted also between luminal A and basal-like samples, and between normal breast-like and basal-like subtypes. SNHG1 expression was lower in luminal A, luminal B, HER2-enriched, and normal breast-like subtypes compared with basal-like, and also lower in normal breast-like compared with luminal B samples. Interestingly, SNHG1 expression was higher in normal breast-like versus luminal A samples. See Supplementary File S5 (Table S4) for details.

Overall, these results may indicate a high level of heterogeneity among various breast cancer subtypes and their expression of snoRNA-related genes.

#### 2.2.2. Associations of Gene Expression and ER/PR/Her2 Status

Bc-GenExMiner v5.2 was used to find associations of ER/PR/Her2 status and RNAseq expression data (TCGA and/or SCAN-B/GSE96058). The data for SCARNA3 and RNU2-1 appeared incomplete (and not significant) and are not presented.

SCARNA2 and SNORD94 were significantly upregulated in ER-positive and PR-positive tumors, with SCARNA2 also elevated in HER2-negative cases, suggesting a potential link to hormone-responsive, less aggressive breast cancer subtypes. In contrast, SNORD15B and SNHG1 showed decreased expression in ER-positive and PR-positive tumors, with no significant differences based on HER2 status. SNORA68 levels were lower in PR-positive and HER2-negative tumors, while its expression did not significantly differ concerning ER status. See Supplementary File S3 for details.

#### 2.2.3. Associations of Gene Expression and Survival

The impact of gene expression on survival was further assessed using both DNA microarray and RNAseq data via bc-GenExMiner v5.2 and Kaplan–Meier survival analyses.

Based on DNA microarray transcriptomic data, SNORD15B was negatively associated with survival: higher expression correlated with worse overall survival (HR 1.16, *p* = 0.0060) and disease-free survival (HR 1.18, *p* = 0.0017). Conversely, RNAseq data revealed a positive association for SCARNA2, where higher expression was linked to better overall and disease-free survival (both HR 0.68, *p* = 0.0007). This finding is consistent with our experimental data.

### 2.3. Expression of the Investigated Small Non-Coding RNAs in Plasma

In the plasma experiment, five small non-coding RNAs (RNU2-1, SCARNA2, SCARNA3, SNORA68, SNORD15B) were selected based on the results obtained from tissue experiments (differential expression, Ct values and availability of RNA/cDNA) to assess their expression using an independent sample set. NST breast cancer-associated samples (*n* = 21) and healthy control samples (*n* = 20) of plasma were analyzed. The aim was to evaluate the feasibility of detecting RNA of these RNAs in plasma and to explore their potential differential expression compared with healthy controls and/or associations with clinicopathological features, as such data are currently lacking.

ROC analysis revealed that the diagnostic potential of these genes may be limited in this study. The data did not yield sufficient AUC values and either sensitivity or specificity was compromised in all investigated RNAs in these cell-free samples. This may indicate that this RNA fraction might be potentially affected in plasma, limiting its diagnostic utility in distinguishing between patient and control samples. A significant difference between pathological and control samples was observed only for RNU2-1 (−1.5-fold, *p* = 0.03715) while a trend toward increased levels was noted for SCARNA2 and SCARNA3. See Supplementary File S6 (Tables S5 and S6) for details.

Next, we analyzed potential expression differences across pathological plasma samples to determine whether their informational value is enhanced within the disease context. No association was found with tumor or clinical stage. When comparing grade 1 to grade 2, SCARNA2 showed significantly higher expression in grade 2 (*p* = 0.0333), with a trend toward higher expression observed for RNU2-1 (*p* = 0.0938). Similarly, non-significantly higher levels of SCARNA3 (*p* = 0.2215) and SNORD15B (*p* = 0.1891) were seen in grade 2 compared to grade 1. Expression levels of RNU2-1, SCARNA2, and SCARNA3 were also higher in grade 3 compared with grade 1 (all *p* = 0.1385).

Comparing luminal A and B samples, a significant association was observed for SCARNA2, which showed higher expression in luminal B samples (*p* = 0.0207). A similar, non-significant trend of increased expression in luminal B samples was observed for SCARNA3 (*p* = 0.1357) and SNORD15B (*p* = 0.1007).

We also identified a significant association between higher levels of SCARNA2 and Ki-67 positivity at both the 15% (*p* = 0.0207) and 20% (*p* = 0.0393) cut-offs. Expression levels of RNU2-1, SCARNA3, and SNORD15B were also higher in Ki-67-positive samples at both cut-offs, although these differences did not reach statistical significance.

The abovementioned results appear to contradict those obtained from tissue samples, highlighting the need for follow-up studies to clarify the biological or methodological reasons behind this difference between the tissues and plasma.

No association was observed with PR positivity. As all samples were ER-positive and Her2-negative, potential associations with ER/Her2 status could not be assessed. Concerning survival, no recurrences were observed for the patients during 321-week follow-up, while one patient’s death was associated with low expressions of all tested genes.

Finally, we explored possible correlations between the expression levels of the investigated genes and U6 snRNA, miR-451a, and miR-548b-5p, previously quantified in the same plasma samples in our earlier study [5]. A significant correlation was found between SCARNA3 and miR-451a (*r* = 0.4655, *p* = 0.0386).

## 3. Discussion

Identification of novel candidate biomarkers for clinical use in diagnosis, prognosis, or prediction of treatment response remains a major challenge in breast cancer research. Diagnosis of this disease is still primarily based on the mammographic screening and/or ultrasonography [19].

Various sample types have been used to identify such biomarkers. Tumor tissues serve as the primary source of information on the pathological processes involved in breast carcinogenesis, allowing the identification of dysregulated genes. These include both protein-coding genes and their regulatory counterparts, non-coding RNAs of various classes. The interactions among these genes form complex networks targeting fundamental cellular processes. Activation or suppression of genes involved in cell division, proliferation, apoptosis, or metastatic spread ultimately determines whether pathological processes linked to carcinogenesis are halted or, conversely, whether cancer progresses [20].

Genes that promote pathological processes leading to uncontrolled cell division and growth are generally referred to as oncogenes. These can arise from proto-oncogenes through genetic alterations such as mutations or amplifications. In contrast, tumor suppressor genes exert the opposite effect by preventing carcinogenesis [21]. This principle also applies to regulatory molecules, including non-coding RNAs such as microRNAs (miRNAs). Oncogenic miRNAs can suppress the expression of protein-coding tumor suppressors, while tumor suppressor miRNAs can downregulate protein-coding oncogenes [22].

The function of genes can be affected even before they are translated into functional proteins or expressed as regulatory RNA molecules. In addition to direct genetic alterations such as mutations, amplifications, and deletions, epigenetic modifications also play a crucial role. For example, DNA methylation of CpGs at the promoters of genes and histone deacetylation can lead to gene silencing, whereas histone acetylation is generally associated with gene activation. Aberrant methylation patterns characterized by promoter hypermethylation of tumor suppressor genes (such as BRCA1, CDH1, PTEN, RARB) leads to their suppression, whereas global hypomethylation activates oncogenes and promotes genomic instability [23,24].

When such alterations and dysregulations involve both protein-coding and non-coding RNAs, the resulting impact on cellular processes becomes highly complex and depends on multiple factors. This complexity may help explain why certain genetic mutations increase the risk of cancer development without inevitably leading to the disease in many cases. These factors also contribute to variations in gene expression, often making it difficult to classify specific genes strictly as tumor suppressors or oncogenes, as many genes may exert different functions based on the different conditions. The dynamics and potentially dual roles of non-coding RNAs are considered a double-edged sword in breast cancer [25]. Moreover, as we have shown recently, many genes exhibit different expression patterns across various breast cancer subtypes [9]. Therefore, it should not be concluded generally whether a gene is an oncogene or tumor suppressor solely based on the expression differences, as its biological roles may be more complex. On the other hand, if its expression patterns are consistently confirmed through multiple direct and indirect indications, its use as a specific biomarker for different patient subgroups may become feasible.

In the present study, we focused specifically on snoRNA-related genes and one snRNA gene (RNU2-1) in breast cancer. Small nucleolar RNAs (snoRNAs) are important non-coding RNAs with diverse functions primarily involved in the biogenesis and assembly of functional ribosomes. They participate in rRNA modification and processing, and may also exert regulatory functions, thereby influencing the expression of other genes, including oncogenes and tumor suppressors. As a result, snoRNAs can become associated with the processes of carcinogenesis. Indeed, growing evidence suggests that snoRNA-related genes may contribute to breast cancer [14].

However, current knowledge of their presumed roles is based on a relatively limited number of studies. Therefore, we aimed to conduct a more comprehensive investigation of this group of non-coding RNAs using clinical samples from patients with invasive breast cancer. Our findings provide a foundation for a deeper understanding of their complex roles in breast cancer, although some questions remain to be addressed in future research.

Using tumor and benign samples from patients with invasive NST breast cancer, differential expression of snoRNAs and RNU2-1 snRNA was first examined in a screening phase, which identified numerous significantly dysregulated genes. Selected candidate genes were then validated in a subsequent phase. In this validation, five genes (*RNU2-1*, *SCARNA2*, *SNORD15B*, *SCARNA3*, and *SNORD94*) were confirmed to be significantly downregulated in tumor samples, while two genes (*SNORA68* and *SNHG1*) were upregulated compared with benign samples. The potential of these genes to distinguish between tumor and benign samples was further assessed using ROC analysis, revealing favorable AUC values, sensitivity, and specificity for all genes, except for *SNHG1* and *SNORD94*, which showed suboptimal diagnostic performance.

To further support the potential functional roles of the investigated genes, presumed primarily to act as tumor suppressors when downregulated in tumor samples and as oncogenes for the two upregulated genes, we analyzed their associations with clinicopathological data. Here, we assume that higher expression in more advanced stages, higher grades, multifocal disease, lymph node metastasis-positive samples, and Ki-67-positive samples represents an unfavorable, pro-cancerous (oncogenic) factor, whereas lower expression under these conditions may indicate tumor suppressor roles. As noted above, the biological complexity may be more complicated, and the correlations may not be causative. Therefore, we further discuss the results also within the context of other available investigations.

Staging data supported the presumed tumor suppressor roles of SCARNA2, SNORD15B, and RNU2-1, showing their higher expression in early-stage samples compared with more advanced stages. Furthermore, when comparing early- and advanced-clinical-stage groups, these three genes were significantly upregulated in early stages, with a similar trend also observed for SCARNA3 and SNORD94. The data also supported the presumed tumor suppressor role in relation to tumor grade for SCARNA3 and SNORD94, with a partial trend observed for SCARNA2 and SNORD15B.

The significantly lower expression of RNU2-1 in lymph node metastasis-positive samples, along with a decreasing trend observed for SCARNA2, SNORD15B, and SNORD94, as well as the reduced expression of SNORD15B in multifocality-positive samples, all support the presumed tumor suppressor roles of these genes.

A trend toward downregulation was observed in Ki-67-positive samples for SCARNA2, and partially for SNORD94 and SCARNA3. When comparing luminal A and luminal B subtypes, a consistent trend toward reduced expression in luminal B samples was observed for RNU2-1, SCARNA2, SCARNA3, and SNORD15B, in line with expectations. SNHG1 expression was negatively associated with ER positivity, PR positivity, and combined ER+/PR+ status, consistent with its observed upregulation in tumor samples.

Finally, survival data provided additional support for the proposed roles of several genes. Specifically, high expression of SCARNA2 and SNORD94 was associated with favorable progression-free survival, with all recurrence events occurring in the low-expression group. Similarly, four out of five progression events for RNU2-1 and SCARNA3 were noted in the low-expression group, indicating a comparable trend despite lacking statistical significance. We also observed a trend toward improved overall survival in the high-expression group for RNU2-1, SCARNA2, SCARNA3, and SNORD94. In contrast, SNORA68 showed the opposite pattern, with worse overall survival in the high-expression group. Altogether, our data strongly support the notion that SCARNA2 and SNORD94 may function as tumor suppressors in invasive breast cancer. SNORD15B, SCARNA3, and RNU2-1 snRNA also appear to have tumor-suppressive potential, whereas the evidence for oncogenic roles of SNHG1 and SNORA68 was less convincing.

The potential of liquid biopsy was assessed using an independent set of plasma samples from patients with invasive NST breast cancer. However, the tested RNAs, RNU2-1, SCARNA2, SCARNA3, SNORA68, and SNORD15B, did not perform well in ROC analysis. This suggests that optimization of the analytical procedure or the use of alternative sample types (e.g., whole blood or PBMCs) may be necessary, as the representation of these genes’ transcripts in plasma, a cell-free sample type, may be insufficient or may not fully reflect the disease state in breast cancer.

Nevertheless, some findings based on plasma samples did not align with tissue-based results. For example, SCARNA2 showed higher expression in grade 2 compared to grade 1 tumors, higher levels in luminal B than luminal A samples, and an association with Ki-67 positivity. These discrepancies may reflect the existence of compensatory mechanisms elevating circulating SCARNA2 levels. However, the underlying causes and sources of these observations remain unclear and require further investigation.

To compare our findings with external datasets, we utilized bc-GenExMiner v5.2 and multiple data sources. Based on TCGA + GTEx data, lower expression levels of SCARNA3, SNORD94, SNORD15B, and, notably, SNHG1 were observed in tumor samples compared to healthy controls. However, no significant differences were detected between tumor and adjacent normal tissues. Interestingly, the expression pattern for SCARNA2 differed depending on the dataset. In DNA microarray transcriptomic data, SCARNA2 expression was lower in luminal A tumors compared to the basal-like subtype, whereas RNA-seq data showed the opposite trend. Overall, the results across these external datasets were heterogeneous for the genes and breast cancer subtypes examined, complicating interpretation. Regarding patient outcomes, SNORD15B appeared to be a negative prognostic factor for overall survival (OS) and disease-free survival (DFS) according to microarray data. In contrast, RNA-seq data supported SCARNA2 as a favorable prognostic indicator, where higher expression was associated with improved OS and DFS, consistent with our findings.

Concerning the literature on the investigated genes, the following section provides a brief summary of current knowledge. One of the most prominent and promising snoRNA-associated genes in this study was SCARNA2. Although it could be considered a potential tumor suppressor in breast cancer based on several aforementioned indications and associations, further experimental work would be necessary to confirm this.

In cellular homeostasis, reparation of DNA double-strand breaks is carried out by two main pathways, i.e., non-homologous end-joining (NHEJ) or homologous recombination (HR). Bergstrand et al. [26] performed a comprehensive study on SCARNA2 and found that more than 90% of SCARNA2 is associated with chromatin and accumulates at DNA breaks, where it supports initiation of HR by the MRN (MRE11, RAD50 and NBS1) complex. SCARNA2 also plays a functional role in DNA repair by inhibiting DNA-dependent protein kinase (DNA-PK) [18]. The activity of SCARNA2 in DNA repair could thus be generally considered a tumor suppressor role (if further confirmed), similar to the BRCA1 tumor suppressor gene [27].

However, it should be noted that these mechanisms may have also opposite effects in cancer development or in drug resistance. Chen et al. [28] reported that SCARNA2 was identified as the most enriched ATR-binding lncRNA and determined the effect of SCARNA2 on cellular sensitivity to DNA-damaging reagents and investigated the effects of SCARNA2 on radiotherapy in rectal-cancer-patient-derived organoids and xenograft preclinical models. Knockdown of SCARNA2 was used to increase the sensitivity of cancer cells to multiple kinds of DNA damage-related chemoradiotherapy and improved the effects of radiotherapy on patient-derived organoids and xenograft models. Escuin et al. [14] showed SCARNA2 to be downregulated in luminal B compared to luminal A breast cancer tumors. This also supports potential tumor suppressor roles of SCARNA2 in breast cancer. However, these categories can be relative, as the same mechanisms might help cancer cells survive DNA damage. Given the similarity to the effects observed with PARP inhibitors in BRCA-mutated breast cancer, it would be interesting to explore the therapeutic potential of SCARNA2 (and its inhibitors) in this context. Logan et al. [29] suggested that SCARNA2 (and SCARNA9) may form part of a signaling system optimizing rRNA modifications to produce ribosomes best adapted to stress conditions. This underlines the complex roles of these genes.

We repeatedly observed evidence supporting a tumor suppressor role of SNORD94 in the present study. To avoid confusion, SNORD94 is also known as U94, but it is not identical with the viral U94, the latency gene of human herpesvirus 6 (HHV-6). Currently, knowledge of this snoRNA in breast cancer is limited. In a recent RNAseq study on early-stage breast cancer [14], SNORD94 was found to be downregulated in tumors from patients with locoregional metastasis compared to metastasis-negative samples. However, SNORD94 was not included in the snoRNA signature used to distinguish patients with locoregional metastasis and to predict patient outcomes, and other data remain variable making definitive conclusions on its role difficult. In other cancers, SNORD94 has been reported as downregulated in secondary plasma cell leukemia (sPCLs) [30] and in pancreatic adenocarcinoma [31].

SNORD15B also emerged as a candidate tumor suppressor. Similar to SNORD94, it was downregulated in tumors from patients with locoregional metastasis [14]. Evidence regarding its function in breast cancer remains scarce; however, it has been suggested as an oncogene in endometrial cancer [32] and also in colorectal cancer [33]. Recently, Xu et al. [34] focused on SNORD15B in the intestinal epithelium and identified its high expression in intestinal stem cells (ISCs). SNORD15B was shown to interact with interleukin enhancer-binding factor 2 (ILF2) to recruit splicing factors, leading to activation of the Wnt/β-catenin signaling pathway and to ISC stemness maintenance and intestinal regeneration. Participation in this process could be considered a pro-cancer activity of this gene.Considering that SNORD15B appeared as a negative prognostic factor in microarray-based survival analyses, its functional role may be complex and warrants further investigation.

SCARNA3 was also considered a candidate tumor suppressor. Liang et al. [35] examined sentinel lymph node (SLN)-positive breast cancer patients, stratifying them according to non-sentinel lymph node (NSLN) status into positive and negative groups. SCARNA3 was among the 11 most highly expressed RNA genes in the NSLN-negative group. However, additional reports on SCARNA3 remain lacking.

RNU2-1 (U2 snRNA) is a small nuclear RNA involved in the study and was also proposed as a tumor suppressor. Functionally, it binds to the intron branch site and is important in pre-mRNA splicing. As we have previously identified another small nuclear RNA, U6 snRNA, as an important biomarker for breast cancer in both tumor and plasma samples [5,8], we included RNU2-1 in this study as a complementary marker for which evidence remains limited. In lung cancer, levels of several tumor-educated platelet small nuclear RNAs, including RNU2-1, were significantly decreased, and this downregulation was correlated with cancer progression [36], supporting its proposed tumor suppressor role, consistent with our observations.

Two genes (SNHG1 and SNORA68) were evaluated as candidate oncogenes. Xiong et al. [37] demonstrated that SNHG1 is overexpressed in breast cancer and that its tissue expression correlates with ER/PR-negative status and advanced clinical stage. Numerous recent studies have further confirmed its involvement in oncogenic processes. For example, Zhang et al. [38] reported SNHG1 not only with increased expression in breast cancer tissues and cells, but it was also found to be associated with reduced patient survival. Experimental silencing of SNHG1 reduced the proliferative, migratory, and invasive activity of breast cancer cells [38]. Moreover, SNHG1 has been detected in exosomes; exosomes derived from hypoxic breast cancer cells were shown to promote proliferation, migration, and angiogenesis in human umbilical vein endothelial cells (HUVECs) [39]. In line with these findings, Deng et al. [40] demonstrated that SNHG1 downregulation suppressed proliferation, migration, invasion, and epithelial–mesenchymal transition (EMT) in breast cancer cells, with miR-641 identified as its target. Beyond breast cancer, SNHG1 has also been described as an oncogene in several other cancers, including colorectal [41], prostate [42], and bladder cancer [43].

Evidence for SNORA68 is more limited. Zhang et al. [44] reported its overexpression in triple-negative breast cancer (TNBC), where it correlated with tumor size, Ki-67 expression, and TNM stage. High SNORA68 expression was associated with significantly shorter disease-free survival. Furthermore, plasma SNORA68 levels were significantly lower in patients who achieved clinical benefit compared with those who did not [44]. 

Despite significant progress, challenges remain in unraveling the biological complexity of snoRNAs and translating these insights into clinical applications. Nevertheless, their potential as diagnostic and prognostic biomarkers in breast cancer and across various diseases and the complexity of their biological roles warrants further investigation [45].

## 4. Limitations of the Study

We performed the present study to shed more light on the role of small nucleolar RNAs in breast cancer. As a discovery phase study focusing on the potential clinical relevance of the investigated genes, we selected a subset of the snoRNA family (along with RNU2-1 snRNA) and explored their expression in tissue and plasma sample sets. Other snoRNA-related genes should be explored as well to achieve a more comprehensive coverage of this group. The number of patient samples available for this study was limited; this was partly compensated by the inclusion of external datasets, which enhanced the robustness of the study, but they also carry their own limitations. The results of the plasma analyses were only tentative, as the expression levels were generally low and ROC analysis indicated a limited diagnostic strength. It may suggest that methodology for plasma isolation and further processing should be more optimized or that the variation in the gene expression is not strongly associated with the disease. We also suggest investigations using other body fluids (whole blood, serum, urine) to evaluate potential and feasibility of snoRNAs as the diagnostic or prognostic biomarkers in breast cancer. The limited number of available studies may indicate that this field remains underexplored both from clinical or experimental perspectives. However, the potential of small nucleolar RNAs appears promising.

## 5. Material and Methods

### 5.1. Patients and Samples

#### 5.1.1. Part I—Tumor and Benign Tissues

Tumor samples (*n* = 22) in the tissue experiment were obtained from patients undergoing surgery for invasive breast carcinoma of no special type (NST, i.e., invasive ductal carcinoma) at the University Hospital in Brno (FN Brno). Benign tissue samples (*n* = 20) were collected either from the same patients or from other breast cancer patients. These benign samples represented mammary tissue with normal histological structure, without any signs of invasive or in situ carcinoma. Tumor and benign tissue samples were preserved in RNAlater solution (Thermo Fisher Scientific, Waltham, MA, USA; Baltics UAB, Vilnius, Lithuania) immediately after collection and histopathological assessment to ensure RNA stability. None of the patients had received chemotherapy or hormonal treatment at the time of tissue collection. The pathological assessment included the determination of levels of progesterone (PR) and estrogen receptors (ERs), Ki-67 proliferation marker, and human epidermal growth factor receptor 2 (Her2) by standard immunohistochemistry. The following antibodies were used in this analysis: Her2—Ventana (4B5), progesterone receptor (PR)—Ventana (Roche Diagnostics, Indianapolis, IN, USA), estrogen receptor (ER)—Zytomed Systems, Ki-67 (clone MIB-1, Zeta Corporation). Luminal A samples were ER+PR+ (≥1%), Her2-negative and Ki67-low (<15%). Luminal B (=luminal B, Her2-negative) samples were ER+ (≥1%), PR-negative or low, Her2-negative and Ki-67-high (≥15%). The cut-off applied in association analyses (e.g., 15% for Ki-67) means that samples were classified as positive if ≥15% of the analyzed cells showed positivity in histopathological assessment. The same principle was used for ER and PR cut-offs.

#### 5.1.2. Part II—Blood Plasma

In the plasma experiment, breast cancer patients diagnosed with NST breast carcinoma were enrolled at the Institute for the Care of Mother and Child (ÚPMD Prague—Podolí) and provided blood samples (*n* = 21) collected into cf-DNA/cf-RNA Preservative Tubes (Norgen Biotek, Thorold, ON, Canada, cat. no. 63950). Control blood samples from healthy female donors (*n* = 20) were collected in the same way at the General University Hospital in Prague (VFN Praha, Faculty/Blood transfusion department/FTO).

All enrolled participants who provided samples for both aforementioned experiments were Caucasian females. Their key clinicopathological characteristics are summarized in Supplementary File S7 (Table S7). The study was approved by the multicenter Ethics Committee of the General University Hospital in Prague (VFN Praha, no. 127/20 S-IV), the Ethics Committee of the University Hospital in Brno (FN Brno), and the Ethics Committee of the Institute for the Care of Mother and Child (ÚPMD Prague—Podolí). The study was conducted in accordance with the Declaration of Helsinki and all participants provided written informed consent.

### 5.2. Selection of the Investigated Genes

In the screening phase (tissue experiment), candidate snoRNAs and snoRNA-related genes were selected based on literature reports suggesting their potential relevance in breast cancer. To cover different classes of snoRNAs, the study included SNORDs, H/ACA box snoRNAs, scaRNAs, SNHGs, and one additional small nuclear RNA, *RNU2-1*, which has been reported as a promising candidate. Based on the screening results, six snoRNA-related genes and *RNU2-1* were further investigated in the validation phase. The selection of genes was made according to the level of differential expression, the consistency across different data processing procedures (Ct < 35 data versus Ct ≤ 40, global mean normalization versus endogenous controls), and statistical significance. For example, *SNORD94* had highest downregulation and along with *RNU2-1* and *SNHG1* were identified as the genes with consistent results across all the procedures, *SNORA68* had the highest upregulation, and *SCARNA3* and *SNORD15B* appeared to have very high downregulation. *SCARNA2* was included as it also had remarkable downregulation, and according to preliminary analyses in bc-GenExMiner v5.2, it was associated with survival outcomes. In the plasma experiment, the same genes have been analyzed except for *SNORD94* and *SNHG1* due to technical limitations concerning available RNA and cDNA. The list of investigated genes and the corresponding TaqMan expression assays is provided in Supplementary File S8 (Table S8).

### 5.3. Total RNA Isolation, Reverse Transcription and qPCR

Total RNA was extracted from tissue samples using mirVana miRNA Isolation Kit, with phenol (Thermo Fisher Scientific, AM1560). In this process, tissue samples were mechanically homogenized using Tissue Ruptor (Qiagen, Hilden, Germany). Collected blood samples were centrifuged at 430× *g* for 20 min at RT and then at 2500× *g* for 10 min at 4 °C to obtain plasma. Total RNA from 2 mL of plasma was isolated using Plasma/Serum Circulating and Exosomal RNA Purification Maxi Kit (Slurry Format, Norgen Biotek, cat. no. 50900). Isolated RNA was stored at −80 °C.

Total RNA from tissue samples was reverse-transcribed using SuperScript IV VILO Master Mix (Thermo Fisher Scientific, 11766050) following ezDNase treatment. Total RNA extracted from plasma samples was reverse-transcribed using the LunaScript RT SuperMix Kit (NEB, Ipswich, MA, USA, E3010). Screening qPCR (tissues): Custom TaqMan Array Fast Plates (Thermo Fisher Scientific, 4413255), containing 2 × 42 selected assays and 2 × 6 endogenous controls, were used in the screening phase. One plate was used for two samples, with 10 µL reaction volumes per gene. For the validation phase (tissues, plasma), expression of each investigated gene was analyzed in duplicate and TaqMan Fast Advanced Master Mix for qPCR (Thermo Fisher Scientific, 4444557) was used as the reaction master mix.

The QuantStudio 3 System (Thermo Fisher Scientific) was used for qPCR analysis. The following fast-mode thermal cycling conditions were applied: 50 °C for 2 min, 95 °C for 20 s, followed by 40 cycles of 95 °C for 1 s and 60 °C for 20 s.

### 5.4. Statistical Analysis

qbase+ software, version 3.4 (Biogazelle/CellCarta, Ghent, Belgium) [46,47] was used for processing expression data and calculating fold differences. The results were corrected for multiple testing using the Benjamini–Hochberg procedure. The geNorm algorithm (implemented in qbase+) was applied to evaluate candidate endogenous controls.

In the screening phase (tissue experiment), two normalization procedures were applied. First, six endogenous controls (*GAPDH*, *IPO8*, *RPLP0*, *18S*, *ACTB*, and *TBP*) were tested, and *GAPDH*, *TBP*, and *RPLP0* were selected as final reference genes based on geNorm analysis. In the validation phase, *GAPDH*, *GUSB*, and *HPRT1* served as endogenous controls, as determined in our recent study [9]. Global mean normalization was used as a secondary strategy in both phases. Data for the plasma samples were normalized by *ACTB* based on the tests. Log-transformed calibrated normalized relative quantities (CNRQs) were exported from qbase+ and further analyzed using MedCalc statistical software, version 20.009, 64-bit (MedCalc Software Ltd., Ostend, Belgium).

Mann–Whitney tests, receiver operating characteristic (ROC) curve analyses to assess the area under the curve (AUC), sensitivity, and specificity, and Kaplan–Meier survival analyses were performed to evaluate differential gene expression and the associations with clinicopathological variables. *p* values < 0.05 were considered statistically significant.

## 6. Conclusions

Small nucleolar RNAs (snoRNAs) have recently emerged as a group of non-coding RNAs with promising potential for clinical application. However, snoRNA research remains limited and current understanding is constrained by heterogeneous and occasionally inconsistent results, methodological differences, and insufficient independent validation. In this study, we evaluated the expression of selected candidate snoRNAs, along with small nuclear RNA gene RNU2-1, in tumor and benign tissues, as well as plasma samples, from Czech population cohorts with invasive NST breast cancer, for which such data have so far been lacking. External validation was carried out using publicly available datasets (e.g., TCGA).

Based on tissue expression data, SCARNA2 was identified as the most promising biomarker (particularly prognostic), likely acting as a tumor suppressor, with supporting evidence from expression patterns, clinicopathological associations and patient outcomes. Additional tumor-suppressive potential was demonstrated for SNORD94 (possibly also prognostic), SNORD15B, SCARNA3, and RNU2-1. In contrast, evidence for the proposed oncogenic roles of SNHG1 and SNORA68, and for the clinical utility of plasma-based detection, was less consistent.

Further research is warranted to assess the diagnostic and prognostic value of selected small nucleolar and small nuclear RNAs and to confirm their potential as biomarkers in breast cancer. If further confirmed, stratifying breast cancer patients based on their expression levels of these and other relevant genes could enable more precise follow-up and ultimately provide clinical benefits for patients. However, the differences observed between breast cancer subtypes may indicate distinct regulatory mechanisms and snoRNA activity within these subtypes, suggesting that their use as biomarkers may not be straightforward. Additionally, further studies should aim to elucidate the biological functions of small nucleolar RNA-related genes in breast carcinogenesis, which may have potential applications in the development of novel targeted therapies. Overall, further research on small nucleolar RNAs and small nuclear RNAs may contribute significantly to advancing the clinical management of breast cancer.

## Figures and Tables

**Table 1:** List of differentially expressed genes and the fold differences between tumor and benign breast tissues (Tissue experiment, validation).

I. Endogenous control normalization					
Downregulated expression					
**Gene**	***p***	**Ratio**	**FD**	**95% CI, Low (FD)**	**95% CI, High (FD)**
*RNU2-1*	0.00008	0.17	−5.83	−11.29	−3.01
*SNORD15B*	0.00070	0.24	−4.10	−8.65	−1.95
*SCARNA2*	0.00021	0.28	−3.57	−6.34	−2.01
*SNORD94*	0.00261	0.33	−3.06	−5.90	−1.59
*SCARNA3*	0.00070	0.34	−2.96	−5.08	−1.72
**Upregulated expression**					
**Gene**	***p***	**Ratio**	**FD**	**95% CI, Low (FD)**	**95% CI, High (FD)**
*SNORA68*	0.00070	3.30	3.30	1.75	6.22
*SNHG1*	0.14896 *	*1.41*	*1.41*	*0.99*	*2.01*
**II. Global mean normalization**					
**Downregulated expression**					
**Gene**	***p***	**Ratio**	**FD**	**95% CI, Low (FD)**	**95% CI, High (FD)**
*RNU2-1*	0.000002	0.22	−4.48	−7.05	−2.85
*SNORD15B*	0.00013	0.32	−3.15	−5.27	−1.89
*SCARNA2*	0.00004	0.36	−2.74	−4.22	−1.78
*SNORD94*	0.00069	0.43	−2.35	−3.78	−1.46
*SCARNA3*	0.00001	0.44	−2.27	−3.13	−1.65
**Upregulated expression**					
**Gene**	***p***	**Ratio**	**FD**	**95% CI, Low (FD)**	**95% CI, High (FD)**
*SNHG1*	0.00008	1.83	1.83	1.42	2.36
*SNORA68*	0.00014	4.29	4.29	2.25	8.19

Notes: *GAPDH*, *GUSB*, and *HPRT1* were used as endogenous controls. FD = fold difference (at both cut-offs, as for Ct ≤ 40 and Ct < 35, the results were identical). FD (y) in downregulated genes was calculated as y = −1/x where x is a ratio value. In upregulated genes, the FD and ratio values are identical. CI-confidence interval. *p* = significance level. * statistically non-significant result.

## Data Availability

All data generated or analyzed during this study are included in this article. Further enquiries can be directed to the corresponding author.

## Supplementary Materials

The following supporting information can be downloaded at: https://www.mdpi.com/article/10.3390/ncrna11060076/s1, Supplementary Files S1–S8.
