# Supplementary material for: Evaluation of Expression and Clinicopathological Relevance of Small Nucleolar RNAs (snoRNAs) in Invasive Breast Cancer
Source: Noncoding RNA. 2025 Oct 31;11(6):76. doi: 10.3390/ncrna11060076 (PMC12642022; doi:10.3390/ncrna11060076)
Supplement: Supplementary file 1 [file ncrna-11-00076-s001.zip › Supplementary file S1.pdf]

Supplementary file S1

Table S1. Differential expression of genes and corresponding fold differences between tumor and benign breast tissues (Tissue experiment, screening)

**I. Global mean normalization**					
**Downregulated expression** (cancer versus control, significant data shaded, non-significant results in italics)					
**Gene/Assay ID**	***p***	**Ratio**	**FD (Ct <35)**	**95% CI, low (FD)**	**95% CI, high (FD)**
SNORD94- Hs03309798_s1	0.00668	0.22	–4.46	–8.20	–2.43
RNU2-1- Hs02786874_gH	0.01336	0.41	–2.45	–3.63	–1.65
SCARNA3;RFWD2-Hs03298706_s1	*0.07053*	*0.23*	*–4.36*	*–10.58*	*–1.80*
SNORD15B-Hs03296837_s1	*0.08079*	*0.43*	*–2.30*	*–4.30*	*–1.23*
SCARNA17-Hs03298712_s1	*0.07053*	*0.44*	*–2.26*	*–4.54*	*–1.12*
SCARNA2-Hs04232660_s1	*0.15321*	*0.49*	*–2.02*	*–3.82*	*–1.07*
SNORD89;RNF149-Hs03298802_s1	*0.07053*	*0.50*	*–2.00*	*–3.26*	*–1.23*
SNORD48-Hs04931161_g1	*0.25484*	*0.52*	*–1.91*	*–4.04*	*–0.90*
SNORD97-Hs03301409_s1	*0.07053*	*0.55*	*–1.81*	*–2.71*	*–1.21*
SCARNA21-Hs03464476_s1	*0.25484*	*0.56*	*–1.80*	*–3.42*	*–0.94*
SCARNA10-Hs03309805_s1	*0.33518*	*0.56*	*–1.77*	*–3.45*	*–0.91*

SCARNA1-Hs03298705_s1	*0.28761*	*0.57*	*–1.74*	*–4.39*	*–0.69*
SCARNA8-Hs03298719_s1	*0.25484*	*0.64*	*–1.56*	*–3.16*	*–0.77*
SNORD16-Hs06626885_s1	*0.51369*	*0.71*	*–1.41*	*–2.45*	*–0.81*
SCARNA28-Hs04937981_s1	*0.87848*	*0.71*	*–1.41*	*–3.01*	*–0.66*
SCARNA4-Hs03298714_s1	*0.25058*	*0.73*	*–1.38*	*–1.97*	*–0.96*
SCARNA7-Hs03309492_s1	*0.25484*	*0.73*	*–1.37*	*–2.01*	*–0.94*
SCARNA5-Hs03298717_s1	*0.62087*	*0.78*	*–1.29*	*–2.35*	*–0.71*
SNHG7;SNORA17B-Hs01075370_g1	*0.51369*	*0.80*	*–1.25*	*–2.66*	*–0.58*
TERC-Hs03297287_s1	*0.81746*	*0.87*	*–1.15*	*–2.65*	*–0.50*
SCARNA11-Hs04333779_s1	*0.38346*	*0.87*	*–1.15*	*–2.06*	*–0.65*
SCARNA6-Hs03298715_s1	*0.64117*	*0.91*	*–1.10*	*–1.93*	*–0.62*
SCARNA14-Hs03309806_s1	*0.81686*	*0.95*	*–1.05*	*–1.77*	*–0.62*
**Upregulated expression** (cancer versus control, significant data shaded, non-significant in italics)					
**Gene/Assay ID**	***p***	**Ratio/FD (Ct <35)**		**95% CI, low**	**95% CI, high**
***SNHG1*-**Hs00411543_m1	0.02673	4.47		2.10	9.55
SNORA68-Hs04416102_s1	*0.06873*	*9.56*		*2.96*	*30.89*

SCARNA27-Hs06633095_g1	*0.08019*	*3.29*	*1.78*	*6.10*
SCARNA22-Hs03298713_s1	*0.14906*	*3.02*	*1.05*	*8.74*
SNHG16-Hs01598403_g1	*0.16509*	*2.60*	*0.92*	*7.37*
SNHG3-Hs05055352_s1	*0.06873*	*2.04*	*1.36*	*3.07*
SCARNA20-Hs04333773_s1	*0.10079*	*2.01*	*0.98*	*4.12*
TBP-Hs00427620_m1	*0.16509*	*1.77*	*1.00*	*3.12*
SNHG15-Hs03301064_g1	*0.28761*	*1.71*	*0.93*	*3.16*
SCARNA23-Hs03309493_s1	*0.48665*	*1.65*	*0.74*	*3.68*
SNHG5-Hs05037597_s1	*0.48665*	*1.63*	*0.76*	*3.50*
SNHG9-Hs03299045_g1	*0.62087*	*1.53*	*0.72*	*3.26*
SCARNA18-Hs04232679_s1	*0.16509*	*1.50*	*0.92*	*2.44*
SNORA71A-Hs03309449_s1	*0.44375*	*1.34*	*0.84*	*2.15*
SNHG11-Hs00290821_m1	*0.56698*	*1.34*	*0.68*	*2.63*
SNHG6-Hs00996619_g1	*0.77497*	*1.25*	*0.54*	*2.89*
SCARNA9-Hs03464471_s1	*0.77497*	*1.20*	*0.49*	*2.91*
SCARNA12-Hs03309494_s1	*0.51369*	*1.15*	*0.63*	*2.07*
SNORD15A-Hs04332088_s1	*0.66678*	*1.08*	*0.42*	*2.78*
SNHG8-Hs03461282_g1	*0.77497*	*1.05*	*0.69*	*1.62*

**Downregulated expression** (cancer versus control, significant data shaded, non-significant in italics)					
**Gene/Assay ID**	***P***	**Ratio**	**FD (Ct≤40)**	**95% CI, low (FD)**	**95% CI, high (FD)**
***SNORD94*-** Hs03309798_s1	0.00653	0.21	–4.80	–9.18	–2.51
***SCARNA3*;RFWD2-** Hs03298706_s1	0.04373	0.21	–4.69	–11.31	–1.95
***RNU2-1*-** Hs02786874_gH	0.01305	0.38	–2.63	–4.02	–1.73
***SNORD15B*-** Hs03296837_s1	0.04373	0.40	–2.47	–4.67	–1.31
SCARNA17-Hs03298712_s1	*0.06677*	0.41	*–2.43*	*–5.00*	*–1.18*
SCARNA2-Hs04232660_s1	*0.10617*	0.46	*–2.18*	*–4.25*	*–1.12*
SNORD89;RNF149-Hs03298802_s1	*0.05637*	0.46	*–2.15*	*–3.74*	*–1.24*
SNORD48-Hs04931161_g1	*0.26969*	0.49	*–2.06*	*–4.64*	*–0.91*
SNORD97-Hs03301409_s1	*0.04196*	0.51	*–1.95*	*–3.08*	*–1.23*
SCARNA21-Hs03464476_s1	*0.26969*	0.52	*–1.93*	*–3.97*	*–0.94*
SCARNA10-Hs03309805_s1	*0.13094*	0.52	*–1.91*	*–3.62*	*–1.01*
SCARNA1-Hs03298705_s1	*0.26969*	0.53	*–1.87*	*–5.01*	*–0.70*
SCARNA8-Hs03298719_s1	*0.26969*	0.60	*–1.68*	*–3.35*	*–0.84*
SNORD16-Hs06626885_s1	*0.35175*	0.66	*–1.51*	*–2.62*	*–0.87*
SCARNA28-	*0.83837*	0.66	*–1.51*	*–3.20*	*–0.71*

Hs04937981_s1					
SCARNA6-Hs03298715_s1	*0.26969*	0.67	*–1.50*	*–2.73*	*–0.83*
SCARNA4-Hs03298714_s1	*0.08438*	0.67	*–1.48*	*–2.11*	*–1.04*
SCARNA7-Hs03309492_s1	*0.26969*	0.68	*–1.48*	*–2.26*	*–0.97*
SCARNA5-Hs03298717_s1	*0.50175*	0.72	*–1.38*	*–2.49*	*–0.77*
SNHG7;SNORA17B-Hs01075370_g1	*0.35175*	0.75	*–1.34*	*–2.66*	*–0.68*
TERC-Hs03297287_s1	*0.65127*	0.81	*–1.24*	*–2.96*	*–0.52*
SCARNA11-Hs04333779_s1	*0.50175*	0.96	*–1.04*	*–1.96*	*–0.55*
SNHG8-Hs03461282_g1	*0.89990*	0.98	*–1.02*	*–1.52*	*–0.69*
**Upregulated expression** (cancer versus control, significant data shaded, non-significant in italics)					
**Gene/Assay ID**	***P***	**Ratio/FD (Ct≤40)**		**95% CI, low**	**95% CI, high**
***SNORA68*-**Hs04416102_s1	0.04196	8.89		2.72	29.08
***SNHG1*-**Hs00411543_m1	0.01566	4.16		2.03	8.54
***SCARNA22*-**Hs03298713_s1	0.04373	4.15		1.51	11.41
***SCARNA27*-**Hs06633095_g1	0.01566	3.97		2.30	6.84
***SNHG3*-**Hs05055352_s1	0.01566	2.78		1.45	5.34
SNHG16-Hs01598403_g1	*0.16048*	*2.42*		*0.89*	*6.56*
SNHG11-	*0.19363*	*2.24*		*0.97*	*5.17*

Hs00290821_m1				
SCARNA20-Hs04333773_s1	*0.26969*	*1.87*	*0.88*	*3.96*
SNHG15-Hs03301064_g1	*0.35175*	*1.59*	*0.90*	*2.83*
SCARNA23-Hs03309493_s1	*0.50175*	*1.53*	*0.68*	*3.44*
SNHG5-Hs05037597_s1	*0.47533*	*1.51*	*0.74*	*3.10*
SCARNA18-Hs04232679_s1	*0.06677*	*1.46*	*1.01*	*2.10*
SNHG9-Hs03299045_g1	*0.65127*	*1.42*	*0.69*	*2.95*
SNORA71A-Hs03309449_s1	*0.56229*	*1.25*	*0.73*	*2.13*
SNHG6-Hs00996619_g1	*0.77636*	*1.16*	*0.54*	*2.52*
SCARNA9-Hs03464471_s1	*0.77636*	*1.11*	*0.48*	*2.60*
SCARNA14-Hs03309806_s1	*0.95913*	*1.07*	*0.56*	*2.05*
SCARNA12-Hs03309494_s1	*0.65127*	*1.07*	*0.55*	*2.06*
SNORD15A-Hs04332088_s1	*0.62427*	*1.01*	*0.39*	*2.58*

**II. Endogenous control normalization** (GAPDH/RPLP0/TBP)					
**Downregulated expression** (cancer versus control, significant data shaded, non-significant in italics)					
**Gene/Assay ID**	***P***	**Ratio**	**FD (Ct <35)**	**95% CI, low (FD)**	**95% CI, high (FD)**
***SNORD94*-**Hs03309798_s1	0.02176	0.09	–10.95	–34.38	–3.49
SCARNA3;RFWD2-	0.02176	0.09	–10.71	–37.73	–3.04

Hs03298706_s1					
RNU2-1- Hs02786874_gH	0.02176	0.17	–6.01	–14.12	–2.56
SNORD15B- Hs03296837_s1	0.02176	0.18	–5.64	–16.58	–1.92
SCARNA17- Hs03298712_s1	0.02670	0.18	–5.54	–17.50	–1.75
SCARNA2- Hs04232660_s1	0.02733	0.20	–4.97	–13.57	–1.82
***SNORD89*;RNF149-** Hs03298802_s1	0.02176	0.20	–4.91	–12.75	–1.89
SNORD48- Hs04931161_g1	0.04823	0.21	–4.69	–14.73	–1.49
SNORD97- Hs03301409_s1	0.02176	0.23	–4.44	–10.46	–1.89
SCARNA21- Hs03464476_s1	0.02733	0.23	–4.41	–13.72	–1.42
SCARNA10- Hs03309805_s1	0.02176	0.23	–4.36	–10.75	–1.77
SNORD16- Hs06626885_s1	0.02670	0.29	–3.45	–8.93	–1.33
SCARNA28- Hs04937981_s1	0.04823	0.29	–3.45	–9.22	–1.29
SCARNA4- Hs03298714_s1	0.02176	0.30	–3.38	–6.95	–1.64
SCARNA7- Hs03309492_s1	0.02733	0.30	–3.37	–7.80	–1.46
***SNHG7*;SNORA17B-** Hs01075370_g1	0.02176	0.33	–3.06	–5.76	–1.63
SNHG8- Hs03461282_g1	0.02733	0.43	–2.33	–4.33	–1.25
SCARNA1-	*0.06924*	0.23	*–4.27*	*–15.65*	*–1.17*

Hs03298705_s1					
SCARNA8-Hs03298719_s1	*0.05625*	0.26	*–3.83*	*–10.31*	*–1.42*
SCARNA5-Hs03298717_s1	*0.05625*	0.32	*–3.16*	*–7.98*	*–1.25*
TERC-Hs03297287_s1	*0.15192*	0.35	*–2.83*	*–9.18*	*–0.87*
SCARNA11-Hs04333779_s1	*0.07016*	0.40	*–2.53*	*–7.20*	*–0.89*
SNORD15A-Hs04332088_s1	*0.17112*	0.44	*–2.26*	*–8.10*	*–0.63*
SCARNA14-Hs03309806_s1	*0.15162*	0.46	*–2.17*	*–5.19*	*–0.91*
SCARNA12-Hs03309494_s1	*0.23385*	0.47	*–2.14*	*–5.44*	*–0.84*
SCARNA9-Hs03464471_s1	*0.23385*	0.49	*–2.05*	*–5.33*	*–0.79*
SNHG6-Hs00996619_g1	*0.06924*	0.51	*–1.96*	*–3.61*	*–1.07*
SNORA71A-Hs03309449_s1	*0.37256*	0.55	*–1.83*	*–4.42*	*–0.75*
SCARNA6-Hs03298715_s1	*0.17112*	0.55	*–1.82*	*–4.71*	*–0.70*
SNHG9-Hs03299045_g1	*0.15192*	0.62	*–1.60*	*–3.07*	*–0.84*
SNHG5-Hs05037597_s1	*0.27358*	0.66	*–1.51*	*–3.11*	*–0.73*
SCARNA23-Hs03309493_s1	*0.23385*	0.67	*–1.49*	*–3.93*	*–0.56*
SNHG15-Hs03301064_g1	*0.15192*	0.70	*–1.43*	*–2.36*	*–0.87*
SNHG11-Hs00290821_m1	*0.72416*	0.80	*–1.25*	*–2.53*	*–0.62*
SCARNA20-Hs04333773_s1	*0.55856*	0.82	*–1.22*	*–2.95*	*–0.51*

SCARNA18-Hs04232679_s1	*0.72416*	0.89	*–1.12*	*–1.98*	*–0.63*
SNHG3-Hs05055352_s1	*0.60242*	0.94	*–1.07*	*–2.47*	*–0.46*
**Upregulated expression** (cancer versus control, significant data shaded, non-significant in italics)					
**Gene/Assay ID**	***P***	**Ratio/FD (Ct <35)**		**95% CI, low**	**95% CI, high**
SNHG1-Hs00411543_m1	0.02733	1.82		1.17	2.84
SNORA68-Hs04416102_s1	*0.05625*	*3.90*		*1.22*	*12.48*
SCARNA27-Hs06633095_g1	*0.10965*	*1.96*		*1.09*	*3.51*
SCARNA22-Hs03298713_s1	*0.17112*	*1.80*		*0.68*	*4.77*
SNHG16-Hs01598403_g1	*0.60242*	*1.06*		*0.48*	*2.36*
**Downregulated expression** (cancer versus control, significant data shaded, non-significant in italics)					
**Gene/Assay ID**	***P***	**Ratio**	**FD (Ct≤40)**	**95% CI, low (FD)**	**95% CI, high (FD)**
SNORD94-Hs03309798_s1	0.01958	0.09	–10.95	–34.38	–3.49
SCARNA3;RFWD2-Hs03298706_s1	0.01958	0.09	–10.71	–37.73	–3.04
RNU2-1-Hs02786874_gH	0.01958	0.17	–6.01	–14.12	–2.56
SNORD15B-Hs03296837_s1	0.01958	0.18	–5.64	–16.58	–1.92
SCARNA17-Hs03298712_s1	0.02448	0.18	–5.54	–17.50	–1.75

SCARNA2-Hs04232660_s1	0.02572	0.20	–4.97	–13.57	–1.82
SNORD89;RNF149-Hs03298802_s1	0.01958	0.20	–4.91	–12.75	–1.89
SNORD48-Hs04931161_g1	0.04569	0.21	–4.69	–14.73	–1.49
SNORD97-Hs03301409_s1	0.01958	0.23	–4.44	–10.46	–1.89
SCARNA21-Hs03464476_s1	0.02572	0.23	–4.41	–13.72	–1.42
SCARNA10-Hs03309805_s1	0.01958	0.23	–4.36	–10.75	–1.77
SNORD16-Hs06626885_s1	0.02448	0.29	–3.45	–8.93	–1.33
SCARNA28-Hs04937981_s1	0.04569	0.29	–3.45	–9.22	–1.29
SCARNA6-Hs03298715_s1	0.01958	0.29	–3.43	–8.05	–1.46
SCARNA4-Hs03298714_s1	0.01958	0.30	–3.38	–6.95	–1.64
SCARNA7-Hs03309492_s1	0.02572	0.30	–3.37	–7.80	–1.46
SNHG7;SNORA17B-Hs01075370_g1	0.01958	0.33	–3.06	–5.76	–1.63
SNHG8-Hs03461282_g1	0.02572	0.43	–2.33	–4.33	–1.25
SCARNA1-Hs03298705_s1	*0.06370*	0.23	*–4.27*	*–15.65*	*–1.17*
SCARNA8-Hs03298719_s1	*0.05136*	0.26	*–3.83*	*–10.31*	*–1.42*
SCARNA5-Hs03298717_s1	*0.05136*	0.32	*–3.16*	*–7.98*	*–1.25*

TERC-Hs03297287_s1	*0.14685*	0.35	*–2.83*	*–9.18*	*–0.87*
SCARNA11-Hs04333779_s1	*0.05136*	0.42	*–2.38*	*–6.31*	*–0.90*
SNORD15A-Hs04332088_s1	*0.17112*	0.44	*–2.26*	*–8.10*	*–0.63*
SCARNA12-Hs03309494_s1	*0.22121*	0.47	*–2.14*	*–5.44*	*–0.84*
SCARNA14-Hs03309806_s1	*0.12909*	0.47	*–2.13*	*–4.77*	*–0.95*
SCARNA9-Hs03464471_s1	*0.22121*	0.49	*–2.05*	*–5.33*	*–0.79*
SNHG6-Hs00996619_g1	*0.06370*	0.51	*–1.96*	*–3.61*	*–1.07*
SNORA71A-Hs03309449_s1	*0.35345*	0.55	*–1.83*	*–4.42*	*–0.75*
SNHG9-Hs03299045_g1	*0.14685*	0.62	*–1.60*	*–3.07*	*–0.84*
SCARNA18-Hs04232679_s1	*0.22121*	0.64	*–1.57*	*–2.88*	*–0.85*
SNHG5-Hs05037597_s1	*0.25918*	0.66	*–1.51*	*–3.11*	*–0.73*
SCARNA23-Hs03309493_s1	*0.22121*	0.67	*–1.49*	*–3.93*	*–0.56*
SNHG15-Hs03301064_g1	*0.14685*	0.70	*–1.43*	*–2.36*	*–0.87*
SCARNA20-Hs04333773_s1	*0.53063*	0.82	*–1.22*	*–2.95*	*–0.51*
SNHG11-Hs00290821_m1	*0.95913*	0.98	*–1.02*	*–1.88*	*–0.55*
**Upregulated expression (**cancer versus control, significant data shaded, non-significant in italics)					
**Gene/Assay ID**	***P***	**Ratio/FD Ct≤40**		**95% CI, low**	**95% CI, high**

***SNHG1*-Hs00411543_m1**	0.02572	1.82	1.17	2.84
SNHG16-Hs01598403_g1	0.58773	1.06	0.48	2.36
SNHG3-Hs05055352_s1	*0.22121*	*1.22*	*0.54*	*2.73*
SCARNA27-Hs06633095_g1	*0.12909*	*1.74*	*1.08*	*2.79*
SCARNA22-Hs03298713_s1	*0.17112*	*1.82*	*0.65*	*5.07*
SNORA68-Hs04416102_s1	*0.05136*	*3.90*	*1.22*	*12.48*

Notes: FD = fold difference, CI – confidence interval. All data corrected for multiple testing (Benjamini-Hochberg procedure). Fold difference (FD) for downregulated gene expression was calculated using the equation y = -1/x (where x is the “ratio” value calculated by qbase+), which results in negative values to indicate downregulation. Accordingly, a -2-fold change corresponds to an FD/ratio of 0.5. When positive values are required, for example when setting 1 as the comparative mean value for benign samples, the FD can be recalculated using the same equation (y = -1/x), where x is a negative FD value.
