# Supplementary material for: Evaluation of Expression and Clinicopathological Relevance of Small Nucleolar RNAs (snoRNAs) in Invasive Breast Cancer
Source: Noncoding RNA. 2025 Oct 31;11(6):76. doi: 10.3390/ncrna11060076 (PMC12642022; doi:10.3390/ncrna11060076)
Supplement: Supplementary file 1 [file ncrna-11-00076-s001.zip › Supplementary file S2.pdf]

Supplementary file S2

Table S2. Results of the receiver operating characteristics analysis for tumors of invasive breast cancer of no special type (n = 22) and benign samples (n = 20) with respect to gene expression (qPCR, Tissue experiment, validation)

Variable (gene expression)	*SCARNA2*	*SCARNA3*	*SNORD94*	*SNORD15B*	*RNU2-1*	*SNHG1*	*SNORA68*
**Area under the ROC curve (AUC)**	0.845	0.805	0.770	0.807	0.873	0.632	0.805
**Standard Error**	0.0609	0.0697	0.0737	0.0710	0.0578	0.0886	0.0728
**95% Confidence interval [a]**	0.701 to 0.938	0.653 to 0.911	0.615 to 0.886	0.656 to 0.912	0.734 to 0.955	0.469 to 0.775	0.653 to 0.911
**z statistic**	5.671	4.368	3.667	4.322	6.444	1.489	4.181
**Significance level *p* (Area=0.5)**	<0.0001	<0.0001	0.0002	<0.0001	<0.0001	0.1366	<0.0001
**Youden index J**	0.6273	0.6136	0.4636	0.6136	0.7091	0.3045	0.6227
**95% Confidence interval [a]**	0.3526 to 0.8045	0.3591 to 0.8091	0.1803 to 0.6318	0.3442 to 0.8091	0.4143 to 0.8545	0.1258 to 0.4576	0.3727 to 0.8136
**Associated criterion**	≤-0.09932	≤0.08873	≤0.07761	≤0.1062	≤0.1317	>0.06596	>-0.1788
**95% Confidence interval**	≤-0.6052 to ≤0.1754	≤-0.01663 to ≤0.4253	≤-0.2739 to ≤0.6311	≤-0.157 to ≤0.274	≤0.07164 to ≤0.5568	>-0.04547 to >0.3369	>-0.1981 to >0.6396
**Sensitivity (%)**	72.73	86.36	86.36	86.36	90.91	45.45	77.27
**Specificity (%)**	90.00	75.00	60.00	75.00	80.00	85.00	85.00
