# Supplementary material for: Evaluation of Expression and Clinicopathological Relevance of Small Nucleolar RNAs (snoRNAs) in Invasive Breast Cancer
Source: Noncoding RNA. 2025 Oct 31;11(6):76. doi: 10.3390/ncrna11060076 (PMC12642022; doi:10.3390/ncrna11060076)
Supplement: Supplementary file 1 [file ncrna-11-00076-s001.zip › Supplementary file S3.pdf]

Záveský L. et al. Evaluation of Expression and Clinicopathological Relevance of Small Nucleolar RNAs (snoRNAs) in Invasive Breast Cancer

Supplementary file S3

Contents:

1. ER and PR receptor status and *SNHG1* expression between benign and tumor breast tissues (Tissue experiment, validation)
2. ER, PR and Her-2 receptor status in RNAseq data for invasive breast cancer (Breast Cancer Gene-Expression Miner v5.2 (bc-GenExMiner v5.2) [16-18]

1. ER and PR receptor status and *SNHG1* expression between benign and tumor breast tissues (Tissue experiment, validation)

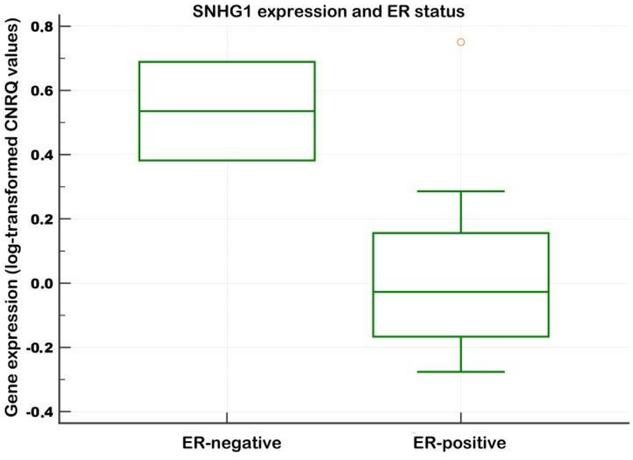

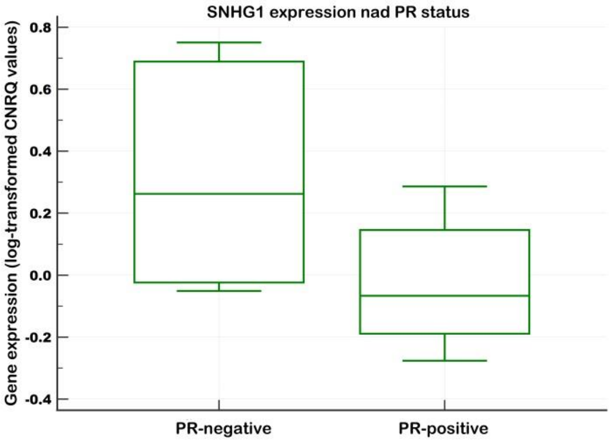

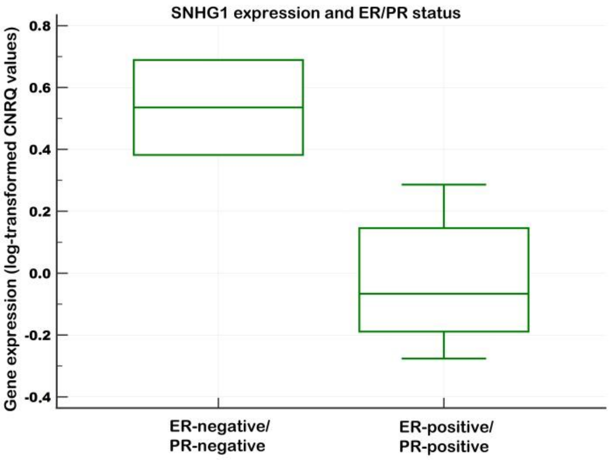

Notes: In Box-and-Whisker plot, a box is drawn from the 1st to 3rd quartile and a horizontal line is drawn at the median. Comparison are based on ER/PR-positivity (10% cut-off applied, i.e. the samples with positivity ≥10% of the analyzed cells by IHC). ER-positive: n = 20, ER-negative: n = 2. PR-positive: n = 16, PR-negative: n = 6. ER-positive/PR-positive: n = 16, ER-negative/PR-negative: n = 2.

2. Gene expression in breast cancer and ER, PR, Her2 receptors

Tables and figures according to RNAseq data by bc-GenExMiner v5.2

1. *SCARNA2* expression (RNAseq data, SCAN-B)

**Summary statistics of *SCARNA2* expression by groups, SCAN-B / GSE96058**							
Groups	Minimum	1st Q	Median	Mean	3rd Q	Maximum	SD
**ER+**	–4.2335	–0.6025	0.0409	0.0116	0.6591	3.59	0.9802
**ER–**	–5.9778	–0.9058	–0.1888	–0.2445	0.4205	3.59	1.1311
**Summary statistics of *SCARNA2* expression by groups**							
Groups	Minimum	1st Q	Median	Mean	3rd Q	Maximum	SD
**PR+**	–4.2335	–0.5978	0.0440	0.0192	0.6559	3.89	0.9700
**PR–**	–5.9778	–0.8218	–0.2409	–0.2019	0.5165	3.59	1.1293
**Summary statistics of *SCARNA2* expression by groups**							
Groups	Minimum	1st Q	Median	Mean	3rd Q	Maximum	SD
**HER2–**	–5.9778	–0.6250	0.0321	0.0032	0.6491	3.89	0.9854
**HER2+**	–3.9528	–0.8334	–0.2359	–0.2548	0.4103	3.59	1.0050

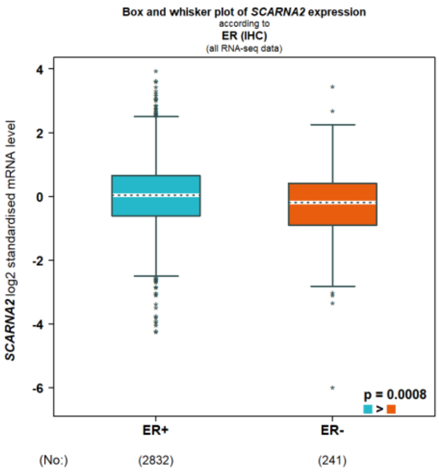

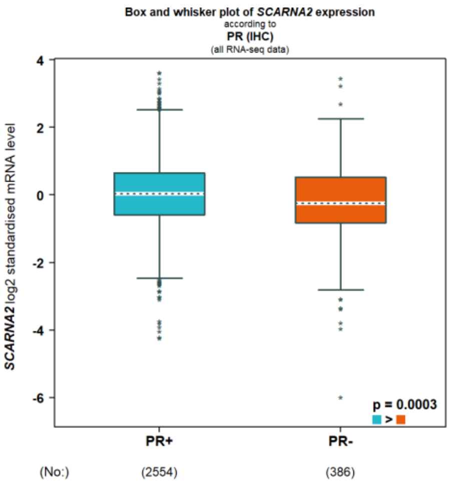

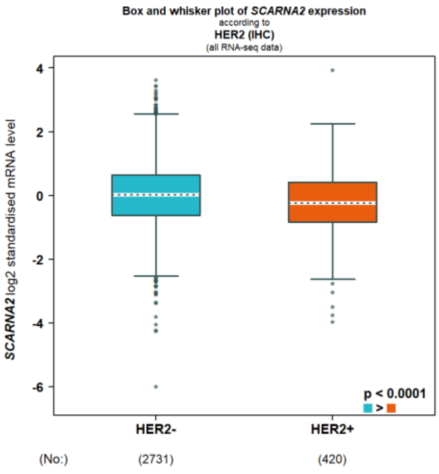

2. *SCARNA3* – not included due to insufficient data in the database

3. *SNORD15B* expression (RNAseq data, TCGA + SCAN-B)

**Summary statistics of *SNORD15B* expression by groups**							
Groups	Minimum	1st Q	Median	Mean	3rd Q	Maximum	SD
**ER+**	–1.8222	–1.2105	–0.0581	–0.2260	0.6135	4.51	1.0094
**ER–**	–1.8222	–1.2105	0.1761	–0.0615	0.7042	3.14	0.9855
**Summary statistics of *SNORD15B* expression by groups**							
Groups	Minimum	1st Q	Median	Mean	3rd Q	Maximum	SD
**PR+**	–1.8222	–1.2105	–0.1059	–0.2315	0.6045	4.51	1.0127
**PR–**	–1.8222	–1.2105	0.1944	–0.0632	0.7182	2.08	0.9903
**Summary statistics of *SNORD15B* expression by groups**							
Groups	Minimum	1st Q	Median	Mean	3rd Q	Maximum	SD
**HER2–**	–1.8222	–1.2105	–0.0430	–0.2383	0.6114	4.51	0.9993
**HER2+**	–1.8222	–1.2105	–0.1470	–0.2351	0.5902	2.44	0.9693

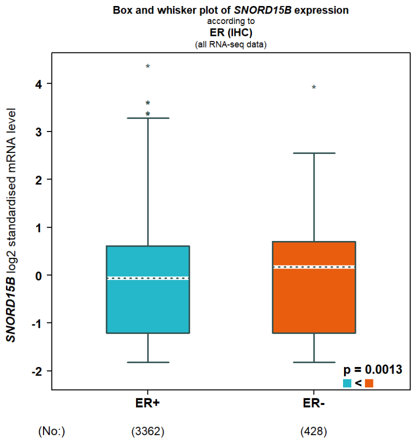

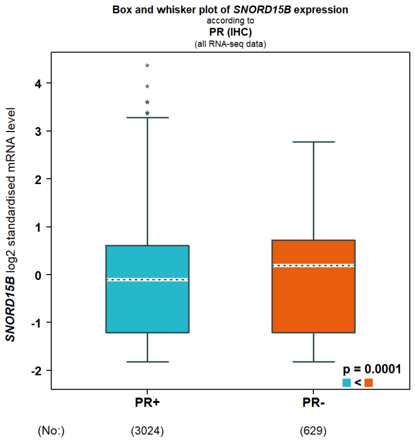

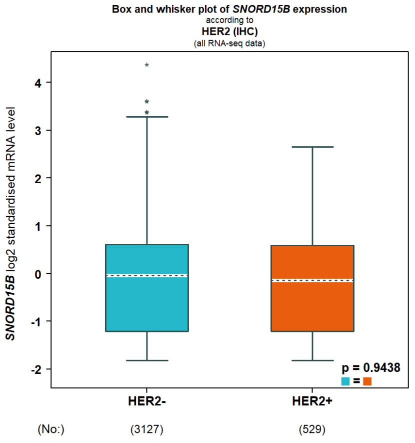

4. *SNORD94* expression (RNAseq data, TCGA + SCAN-B)

**Summary statistics of *SNORD94* expression by groups**							
Groups	Minimum	1st Q	Median	Mean	3rd Q	Maximum	SD
**ER+**	–4.1728	0.0000	0.0000	0.5852	1.79	3.71	1.0490
**ER–**	–4.1728	0.0000	0.0000	0.3440	0.7322	3.57	1.0441
**Summary statistics of *SNORD94* expression by groups**							
Groups	Minimum	1st Q	Median	Mean	3rd Q	Maximum	SD
**PR+**	–4.1728	0.0000	0.0000	0.5861	1.13	3.71	1.0395
**PR–**	–4.1728	0.0000	0.0000	0.3717	0.8972	3.57	1.0888
**Summary statistics of *SNORD94* expression by groups**							
Groups	Minimum	1st Q	Median	Mean	3rd Q	Maximum	SD
**HER2–**	–4.1728	0.0000	0.0000	0.6009	1.14	3.71	1.0275
**HER2+**	–4.1728	0.0000	0.0000	0.5072	1.65	3.57	1.0585

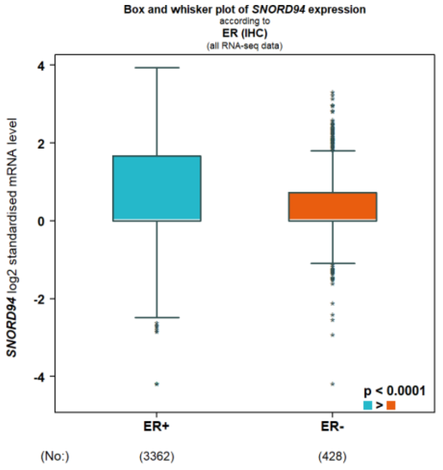

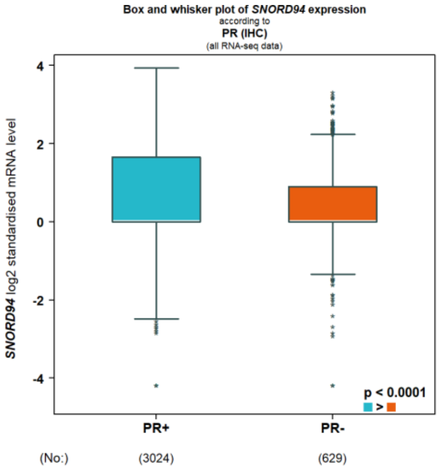

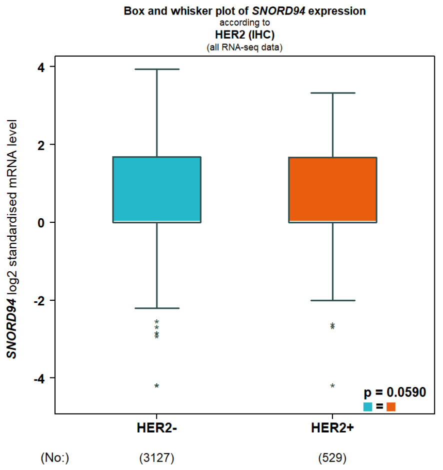

5. *SNORA68* (RNAseq data, SCAN-B)

Summary statistics of *SNORA68* expression by groups							
Groups	Minimum	1st Q	Median	Mean	3rd Q	Maximum	SD
**ER+**	0.0000	0.0000	0.0000	0.8779	1.73	3.23	0.9995
**ER–**	0.0000	0.0000	0.0000	0.9503	1.80	3.1500	1.0179
**Summary statistics of *SNORA68* expression by groups**							
Groups	Minimum	1st Q	Median	Mean	3rd Q	Maximum	SD
**PR+**	0.0000	0.0000	0.0000	0.8647	1.51	3.23	0.9988
**PR–**	0.0000	0.0000	1.37	0.9914	1.91	3.35	1.0180
**Summary statistics of *SNORA68* expression by groups**							
Groups	Minimum	1st Q	Median	Mean	3rd Q	Maximum	SD
**HER2–**	0.0000	0.0000	0.0000	0.8672	1.54	3.23	0.9952
**HER2+**	0.0000	0.0000	0.6401	0.9699	1.28	2.23	0.9938

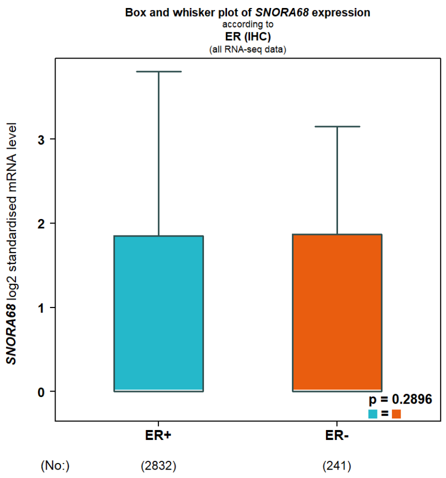

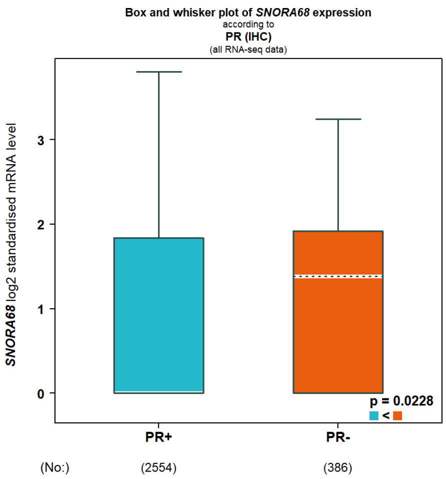

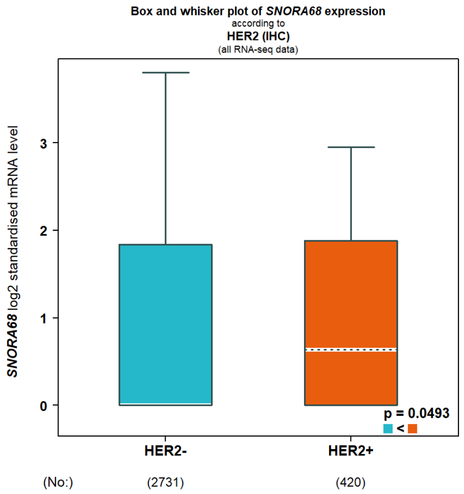

6. *SNHG1* expression (RNAseq data, TCGA + SCAN-B)

**Summary statistics of *SNHG1* expression by groups**							
Groups	Minimum	1st Q	Median	Mean	3rd Q	Maximum	SD
**ER+**	–3.2197	–0.7068	–0.0744	–0.1009	0.5410	5.93	0.9407
**ER–**	–2.6676	–0.2417	0.5055	0.5265	1.74	3.57	1.1239
**Summary statistics of *SNHG1* expression by groups**							
Groups	Minimum	1st Q	Median	Mean	3rd Q	Maximum	SD
**PR+**	–3.2197	–0.7078	–0.0735	–0.1033	0.5340	5.93	0.9403
**PR–**	–2.6676	–0.3396	0.2790	0.3232	1.0528	4.1163	1.0869
**Summary statistics of *SNHG1* expression by groups**							
Groups	Minimum	1st Q	Median	Mean	3rd Q	Maximum	SD
**HER2–**	–3.1691	–0.6755	–0.0188	–0.0138	0.6321	4.1163	1.0000
**HER2+**	–3.2197	–0.7343	0.0364	–0.0593	0.6262	5.93	1.0323

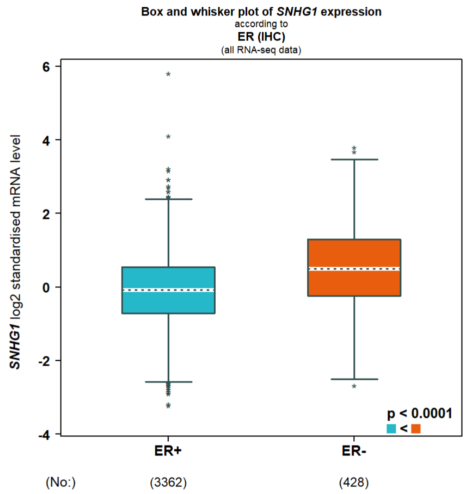

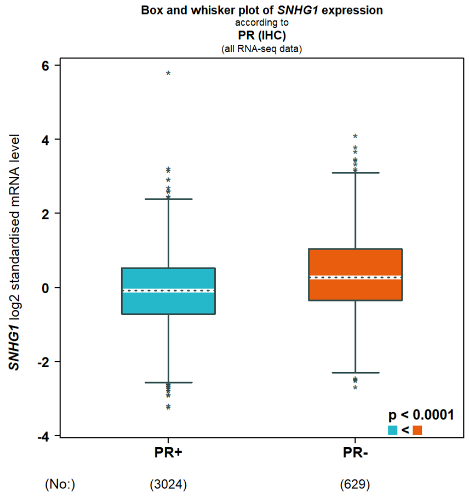

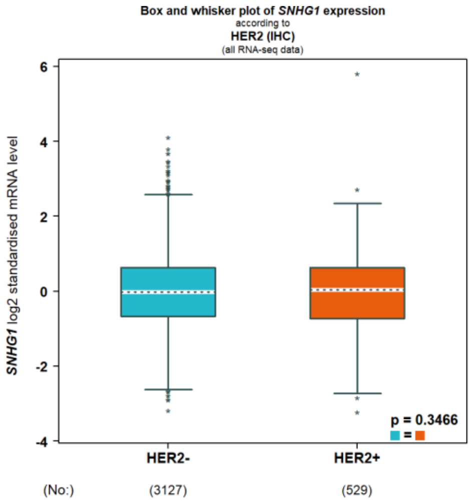

7. *RNU2-1* – not included due to insufficient data in the database

Notes: Data were adopted from Breast Cancer Gene-Expression Miner v5.2 (bc-GenExMiner v5.2) [16-18]
