# Supplementary material for: Evaluation of Expression and Clinicopathological Relevance of Small Nucleolar RNAs (snoRNAs) in Invasive Breast Cancer
Source: Noncoding RNA. 2025 Oct 31;11(6):76. doi: 10.3390/ncrna11060076 (PMC12642022; doi:10.3390/ncrna11060076)
Supplement: Supplementary file 1 [file ncrna-11-00076-s001.zip › Supplementary file S4.pdf]

Supplementary file S4

Table S3. Progression-free survival (PFS) and overall survival (OS) associated with gene expression levels in NST breast cancer patients

A. Progression-free survival (PFS) associated with gene expression levels

**Experiment Tissue, validation**						
**Gene - expression level**	**Mean PFS as calculated in Kaplan-Meier tests (weeks)**	**SE**	**95% CI for the mean**	**Endpoints for PFS**	**Log-rank test for PFS**	**Hazard ratios with [95% Confidence Interval]**
SCARNA2– low expression	272.136	31.198	210.988 to 333.285	5	*P* = 0.0475	*n/a*
SCARNA2– high expression	330.000	0.000	330.000 to 330.000	0		n/a
SCARNA3– low expression	287.636	30.258	228.330 to 346.942	4	*P* = 0.3858	2.3856 [0.3343 to 17.0230]
SCARNA3– high expression	312.667	16.342	280.636 to 344.697	1		0.4192 [0.05874 to 2.9912]
SNORD94– low expression	272.136	31.198	210.988 to 333.285	5	*P* = 0.0475	n/a
SNORD94– high expression	326.000	0.000	326.000 to 326.000	0		n/a

**Experiment Tissue, validation**						
**Gene - expression level**	**Mean PFS as calculated in Kaplan-Meier tests (weeks)**	**SE**	**95% CI for the mean**	**Endpoints for PFS**	**Log-rank test for PFS**	**Hazard ratios with [95% Confidence Interval]**
SNORD15B – low expression	285.727	30.839	225.283 to 346.171	4	*P* = 0.3269	2.6681 [0.3750 to 18.9813]
SNORD15B – high expression	306.111	13.095	280.446 to 331.776	1		0.3748 [0.05268 to 2.6664]
RNU2-1– low expression	287.182	30.409	227.580 to 346.784	4	*P* = 0.3568	2.5154 [0.3536 to 17.8948]
RNU2-1– high expression	313.222	15.818	282.218 to 344.226	1		0.3976 [0.05588 to 2.8283]
SNHG1– low expression	300.364	21.815	257.606 to 343.121	4	*P* = 0.4141	2.2730 [0.3168 to 16.3091]
SNHG1– high expression	300.400	28.081	245.361 to 355.439	1		0.4399 [0.06132 to 3.1567]
SNORA68– low expression	299.300	19.420	261.238 to 337.362	2	*P* = 0.9835	0.9795 [0.1377 to 6.9680]

**Experiment Tissue, validation**						
**Gene - expression level**	**Mean PFS as calculated in Kaplan-Meier tests (weeks)**	**SE**	**95% CI for the mean**	**Endpoints for PFS**	**Log-rank test for PFS**	**Hazard ratios with [95% Confidence Interval]**
SNORA68– high expression	298.500	31.273	237.205 to 359.795	3		1.0210 [0.1435 to 7.2634]

B. Overall survival (OS) associated with gene expression levels

**Experiment Tissue, validation**						
**Gene - expression level**	**Mean OS as calculated in Kaplan-Meier tests (weeks)**	**SE**	**95% CI for the mean**	**Endpoints for OS**	**Log-rank test for OS**	**Hazard ratios with 95% Confidence Interval**
SCARNA2– low expression	303.495	29.353	245.963 to 361.027	2	*P* = 0.1672	n/a
SCARNA2– high expression	330.000	0.000	330.000 to 330.000	0		n/a
SCARNA3– low expression	305.000	28.858	248.439 to 361.561	2	*P* = 0.1913	n/a
SCARNA3– high expression	330.000	0.000	330.000 to 330.000	0		n/a

Experiment Tissue, validation						
**Gene - expression level**	**Mean OS as calculated in Kaplan-Meier tests (weeks)**	**SE**	**95% CI for the mean**	**Endpoints for OS**	**Log-rank test for OS**	**Hazard ratios with 95% Confidence Interval**
SNORD94– low expression	303.495	29.353	245.963 to 361.027	2	*P* = 0.1672	n/a
SNORD94– high expression	326.000	0.000	326.000 to 326.000	0		n/a
SNORD15B– low expression	318.545	27.130	265.370 to 371.721	1	*P* = 0.9731	0.9534 [0.05954 to 15.2680]
SNORD15B– high expression	306.444	12.780	281.395 to 331.494	1		1.0488 [0.06550 to 16.7954]
RNU2-1– low expression	303.495	29.353	245.963 to 361.027	2	*P* = 0.1672	n/a
RNU2-1– high expression	330.000	0.000	330.000 to 330.000	0		n/a
SNHG1– low expression	332.100	14.135	304.395 to 359.805	1	*P* = 0.9103	0.8522 [0.05276 to 13.7652]
SNHG1– high expression	300.400	28.081	245.361 to 355.439	1		1.1734 [0.07265 to 18.9526]

**Experiment Tissue, validation**						
**Gene - expression level**	**Mean OS as calculated in Kaplan-Meier tests (weeks)**	**SE**	**95% CI for the mean**	**Endpoints for OS**	**Log-rank test for OS**	**Hazard ratios with 95% Confidence Interval**
SNORA68– low expression	330.000	0.000	330.000 to 330.000	0	*P* = 0.1474	n/a
SNORA68– high expression	300.800	31.437	239.184 to 362.416	2		n/a

Note: PFS = progression–free survival, OS = overall survival, n/a = not available.
