# Supplementary material for: Evaluation of Expression and Clinicopathological Relevance of Small Nucleolar RNAs (snoRNAs) in Invasive Breast Cancer
Source: Noncoding RNA. 2025 Oct 31;11(6):76. doi: 10.3390/ncrna11060076 (PMC12642022; doi:10.3390/ncrna11060076)
Supplement: Supplementary file 1 [file ncrna-11-00076-s001.zip › Supplementary file S5.pdf]

Supplementary file S5

Table S4. Gene expression according to external datasets processed in bc-GenExMiner v5.2 [16-18]

Gene expression differences based on TCGA and Gtex datasets (bc-GenExMiner v5.2, RNAseq data) for invasive breast cancer, tumor adjacent and normal tissues.

**Summary statistics of *SCARNA3* expression by groups**							
Groups	Minimum	1st Q	Median	Mean	3rd Q	Maximum	SD
**Healthy**	0.0000	0.0000	0.6635	0.6837	1.92	2.03	0.7213
**Tumour-adjacent**	0.0000	0.0000	0.0000	0.2503	0.4222	2.0701	0.4298
**Tumour**	0.0000	0.0000	0.0000	0.2876	0.4461	3.1156	0.4267
**Dunnett-Tukey-Kramer's test:**							
gene-expression comparisons			p-value				
Tumour-adjacent	<	Healthy		< 0.0001			
Tumour	<	Healthy		< 0.0001			
Tumour	=	Tumour-adjacent		> 0.10			

**Summary statistics of *SNORD94* expression by groups**							
Groups	Minimum	1st Q	Median	Mean	3rd Q	Maximum	SD
**Healthy**	2.47	4.40	4.09	4.34	5.10	6.32	0.7566
**Tumour-adjacent**	0.0000	1.05	2.78	2.29	3.0702	4.36	0.7887
**Tumour**	0.0000	1.81	2.54	2.09	2.55	4.14	0.7626
**p(Welch) < 0.0001**							
**Dunnett-Tukey-Kramer's test:**							
gene-expression comparisons			p-value				
Tumour-adjacent	<	Healthy		< 0.0001			
Tumour	<	Healthy		< 0.0001			
Tumour	=	Tumour-adjacent		> 0.10			

**Summary statistics of *SNHG1* expression by groups**							
Groups	Minimum	1st Q	Median	Mean	3rd Q	Maximum	SD
**Healthy**	6.84	7.53	7.34	7.94	7.37	8.84	0.4626
**Tumour-adjacent**	4.00	5.50	6.86	6.1320	6.14	7.21	0.7654
**Tumour**	3.09	5.63	6.1755	6.85	6.92	9.1400	0.8512
**p(Welch) < 0.0001**							
**Dunnett-Tukey-Kramer's test:**							
gene-expression comparisons			p-value				
Tumour-adjacent	<	Healthy		< 0.0001			

Tumour	<	Healthy		< 0.0001			
Tumour	=	Tumour-adjacent		> 0.10			

**Summary statistics of *SNORD15B* expression by groups**							
Groups	Minimum	1st Q	Median	Mean	3rd Q	Maximum	SD
**Healthy**	0.0000	2.0601	2.84	2.43	3.0506	4.74	0.8826
**Tumour-adjacent**	0.0000	0.6767	1.0727	1.1102	1.81	2.97	0.6314
**Tumour**	0.0000	0.5798	1.0490	1.1344	1.65	5.00	0.7893
**p(Welch) < 0.0001**							
**Dunnett-Tukey-Kramer's test:**							
gene-expression comparisons			p-value				
Tumour-adjacent	<	Healthy		< 0.0001			
Tumour	<	Healthy		< 0.0001			
Tumour	=	Tumour-adjacent		> 0.10			

Gene expression differences for different subtypes (bc-GenExMiner v5.2, RNAseq data)

**Summary statistics of *SCARNA2* expression by groups**							
Groups	Minimum	1st Q	Median	Mean	3rd Q	Maximum	SD
**Basal-like**	–5.9778	–0.8126	–0.1292	–0.1533	0.5460	3.59	1.0685
**HER2-E**	–4.2056	–0.9704	–0.3399	–0.3917	0.2521	3.0271	0.9947
**Luminal A**	–3.9009	–0.5367	0.0632	0.0543	0.6180	3.89	0.9146
**Luminal B**	–4.2335	–0.6866	–0.0448	–0.0396	0.6291	3.59	1.0410
**Normal breast-like**	–4.0310	–0.2200	0.3189	0.3093	0.8546	3.27	0.8732
**Dunnett-Tukey-Kramer's test:**							
gene-expression comparisons			p-value				
Luminal A	>	HER2-E		< 0.0001			
Luminal B	>	HER2-E		< 0.0001			
Normal breast-like	>	Basal-like		< 0.0001			
Normal breast-like	>	HER2-E		< 0.0001			
Normal breast-like	>	Luminal A		< 0.0001			
Normal breast-like	>	Luminal B		< 0.0001			
HER2-E	<	Basal-like		< 0.01			
Luminal A	>	Basal-like		< 0.01			
Luminal B	=	Basal-like		> 0.10			
Luminal B	=	Luminal A		> 0.10			

**Summary statistics of *SNORD94* expression by groups**							
Groups	Minimum	1st Q	Median	Mean	3rd Q	Maximum	SD
**Basal-like**	–4.1728	0.0000	0.0000	0.5400	1.45	3.27	1.0499
**HER2-E**	–4.1728	0.0000	0.0000	0.4800	1.81	3.52	1.0218
**Luminal A**	–4.1728	0.0000	0.0000	0.5527	1.29	3.68	1.0644

**Luminal B**	–4.1728	0.0000	0.0000	0.6035	1.38	3.71	1.0717
**Normal breast-like**	–2.2049	0.0000	0.0000	0.6028	1.46	3.87	0.9859

Summary statistics of *SNORD15B* expression by groups

Groups	Minimum	1st Q	Median	Mean	3rd Q	Maximum	SD
**Basal-like**	–1.8222	–1.2105	0.1787	–0.1080	0.7012	3.14	0.9927
**HER2-E**	–1.8222	–1.2105	–0.1605	–0.2502	0.5809	2.84	0.9741
**Luminal A**	–1.8222	–1.2105	–0.3290	–0.2681	0.5580	3.86	1.0023
**Luminal B**	–1.8222	–1.2105	0.0148	–0.2021	0.6166	4.51	1.0208
**Normal breast-like**	–1.8222	–1.2105	0.1257	–0.1791	0.6479	3.86	1.0201

Dunnett-Tukey-Kramer's test:

gene-expression comparisons				p-value
Luminal A	<	Basal-like		< 0.01
HER2-E	<	Basal-like		< 0.10
Luminal A	=	HER2-E		> 0.10
Luminal B	=	Basal-like		> 0.10
Luminal B	=	HER2-E		> 0.10
Luminal B	=	Luminal A		> 0.10
Normal breast-like	=	Basal-like		> 0.10
Normal breast-like	=	HER2-E		> 0.10
Normal breast-like	=	Luminal A		> 0.10
Normal breast-like	=	Luminal B		> 0.10

Summary statistics of *SNORA68* expression by groups

Groups	Minimum	1st Q	Median	Mean	3rd Q	Maximum	SD
**Basal-like**	0.0000	0.0000	0.0000	0.9493	1.63	3.1500	1.0119
**HER2-E**	0.0000	0.0000	0.0000	0.9421	1.52	3.28	0.9992
**Luminal A**	0.0000	0.0000	0.0000	0.8155	1.97	3.11	0.9836
**Luminal B**	0.0000	0.0000	0.0000	0.9010	1.73	3.90	1.0101
**Normal breast-like**	0.0000	0.0000	0.0000	0.8717	1.90	3.23	1.0012

SNHG1

Dunnett-Tukey-Kramer's test:

gene-expression comparisons				p-value
HER2-E	<	Basal-like		< 0.0001
Luminal A	<	Basal-like		< 0.0001
Luminal B	<	Basal-like		< 0.0001
Luminal B	>	Luminal A		< 0.0001
Normal breast-like	<	Basal-like		< 0.0001
Normal breast-like	>	Luminal A		< 0.0001
Luminal A	<	HER2-E		< 0.001
Luminal B	>	HER2-E		< 0.001
Normal breast-like	>	HER2-E		< 0.05
Normal breast-like	=	Luminal B		> 0.10

Summary statistics of *RNU2-1* expression by groups

Groups	Minimum	1st Q	Median	Mean	3rd Q	Maximum	SD
**Basal-like**	0.0000	0.0000	0.0000	0.1685	0.0000	10.1819	1.1106
**HER2-E**	0.0000	0.0000	0.0000	0.1060	0.0000	12.60	0.9326
**Luminal A**	0.0000	0.0000	0.0000	0.0796	0.0000	20.7827	0.8736
**Luminal B**	0.0000	0.0000	0.0000	0.1653	0.0000	17.6654	1.1726
**Normal breast-like**	0.0000	0.0000	0.0000	0.1209	0.0000	8.67	0.9045

Gene expression differences for different subtypes (bc-GenExMiner v5.2, DNA microarray data)

Summary statistics of *RNU2-1* expression by groups

Groups	Minimum	1st Q	Median	Mean	3rd Q	Maximum	SD
**Basal-like**	–2.1282	–0.5363	–0.2134	–0.0915	0.1538	2.97	0.9363
**HER2-E**	–1.5979	–0.3431	–0.0290	–0.1761	0.1033	1.78	0.8108
**Luminal A**	–1.6185	–0.6966	–0.1109	0.0896	0.4584	2.48	1.0856
**Luminal B**	–1.7443	–0.3996	0.2541	0.2168	1.1153	1.56	1.0675
**Normal breast-like**	–1.5051	–0.3802	0.2908	0.3614	0.6249	2.85	1.1274

Summary statistics of *SCARNA2* expression by groups

Groups	Minimum	1st Q	Median	Mean	3rd Q	Maximum	SD
**Basal-like**	–3.1453	–0.6937	0.0767	0.0508	0.7985	2.70	0.9906
**HER2-E**	–3.1862	–0.7935	–0.0109	–0.0884	0.6919	2.42	0.9855
**Luminal A**	–2.9151	–0.8590	–0.0394	–0.1066	0.6250	3.0659	0.9904
**Luminal B**	–3.4800	–0.8267	–0.0377	–0.0678	0.6791	3.1414	1.0119
**Normal breast-like**	–2.9907	–0.6463	0.0905	0.0728	0.7870	2.41	1.0400

Dunnett-Tukey-Kramer's test:

gene-expression comparisons			p-value	
Luminal A	<	Basal-like		< 0.01
Normal breast-like	>	Luminal A		< 0.05
HER2-E	<	Basal-like		< 0.10
Normal breast-like	>	HER2-E		< 0.10
Luminal A	=	HER2-E		> 0.10
Luminal B	=	Basal-like		> 0.10
Luminal B	=	HER2-E		> 0.10
Luminal B	=	Luminal A		> 0.10
Normal breast-like	=	Basal-like		> 0.10
Normal breast-like	=	Luminal B		> 0.10

Summary statistics of *SCARNA3* expression by groups

Groups	Minimum	1st Q	Median	Mean	3rd Q	Maximum	SD
**Basal-like**	–3.0303	–0.4772	0.0231	–0.1446	0.4111	1.82	0.9687
**HER2-E**	–3.0513	–1.4928	–0.3287	–0.6160	0.1039	1.76	1.0412

**Luminal A**	–2.7450	–0.3570	0.1958	0.0445	0.6327	1.46	0.9176
**Luminal B**	–3.2669	–0.7073	–0.1818	–0.3915	0.1970	1.99	0.9571
**Normal breast-like**	–2.9264	–0.5837	0.2905	–0.0046	0.7885	1.28	1.0958

**Dunnett-Tukey-Kramer's test:**			
gene-expression comparisons			p-value
Luminal A	>	HER2-E	< 0.001
HER2-E	<	Basal-like	< 0.05
Luminal B	<	Luminal A	< 0.05
Normal breast-like	>	HER2-E	< 0.10
Luminal A	=	Basal-like	> 0.10
Luminal B	=	Basal-like	> 0.10
Luminal B	=	HER2-E	> 0.10
Normal breast-like	=	Basal-like	> 0.10
Normal breast-like	=	Luminal A	> 0.10
Normal breast-like	=	Luminal B	> 0.10

Summary statistics of *SNORD94* expression by groups

Groups	Minimum	1st Q	Median	Mean	3rd Q	Maximum	SD
**Basal-like**	–2.1132	–0.9973	–0.6156	–0.4831	–0.2877	3.0265	0.9710
**HER2-E**	–2.0887	–0.7230	–0.2152	–0.0684	0.2662	2.09	0.8932
**Luminal A**	–1.3066	–0.2474	0.2844	0.3552	0.9201	2.71	0.9825
**Luminal B**	–1.1592	–0.4814	0.0176	0.2417	0.8789	2.42	1.0010
**Normal breast-like**	–0.7471	0.2626	0.4580	0.6659	0.8186	2.56	0.8845

**Dunnett-Tukey-Kramer's test:**			
gene-expression comparisons			p-value
Normal breast-like	>	Basal-like	< 0.01
Luminal A	>	Basal-like	< 0.05
Normal breast-like	>	HER2-E	< 0.05
Luminal B	>	Basal-like	< 0.10
HER2-E	=	Basal-like	> 0.10
Luminal A	=	HER2-E	> 0.10
Luminal B	=	HER2-E	> 0.10
Luminal B	=	Luminal A	> 0.10
Normal breast-like	=	Luminal A	> 0.10
Normal breast-like	=	Luminal B	> 0.10

Summary statistics of *SNORD15B* expression by groups

Groups	Minimum	1st Q	Median	Mean	3rd Q	Maximum	SD
**Basal-like**	–4.5044	–0.1529	0.3181	0.4454	0.9230	6.00	1.1323
**HER2-E**	–4.0128	–0.3326	0.1488	0.2622	0.7083	4.01	1.0646
**Luminal A**	–4.2179	–0.4972	–0.1189	0.0473	0.4513	8.0216	0.9436
**Luminal B**	–3.5778	–0.4428	0.1562	0.2444	0.7387	7.61	1.1633
**Normal breast-like**	–1.5965	–0.2940	0.2225	0.1748	0.7057	2.24	0.7771

**Dunnett-Tukey-Kramer's test:**	
gene-expression comparisons	p-value

Luminal A	<	Basal-like		< 0.0001
Luminal A	<	HER2-E		< 0.05
Luminal B	>	Luminal A		< 0.05
Normal breast-like	<	Basal-like		< 0.10
HER2-E	=	Basal-like		> 0.10
Luminal B	=	Basal-like		> 0.10
Luminal B	=	HER2-E		> 0.10
Normal breast-like	=	HER2-E		> 0.10
Normal breast-like	=	Luminal A		> 0.10
Normal breast-like	=	Luminal B		> 0.10

Summary statistics of *SNORA68* expression by groups

Groups	Minimum	1st Q	Median	Mean	3rd Q	Maximum	SD
**Basal-like**	–4.0791	–0.6281	–0.1111	–0.1171	0.5049	3.1056	0.9906
**HER2-E**	–3.9855	–0.7674	–0.1432	–0.2038	0.4324	4.52	0.9687
**Luminal A**	–7.4018	–0.4236	0.0620	0.0448	0.7044	2.99	0.9756
**Luminal B**	–3.7621	–0.6324	0.0428	0.0138	0.6454	3.04	1.0152
**Normal breast-like**	–5.1375	–0.3950	0.1286	0.1234	0.8611	3.06	1.0459

Dunnett-Tukey-Kramer's test:

gene-expression comparisons				p-value
Luminal A	>	HER2-E		< 0.001
Normal breast-like	>	HER2-E		< 0.001
Luminal B	>	HER2-E		< 0.01
Luminal A	>	Basal-like		< 0.05
Normal breast-like	>	Basal-like		< 0.05
HER2-E	=	Basal-like		> 0.10
Luminal B	=	Basal-like		> 0.10
Luminal B	=	Luminal A		> 0.10
Normal breast-like	=	Luminal A		> 0.10
Normal breast-like	=	Luminal B		> 0.10

Summary statistics of *SNHG1* expression by groups

Groups	Minimum	1st Q	Median	Mean	3rd Q	Maximum	SD
**Basal-like**	–2.2777	–0.0566	0.5734	0.5691	1.04	3.95	0.9666
**HER2-E**	–3.8358	–0.5082	0.0470	0.0182	0.6119	3.14	0.9268
**Luminal A**	–3.6771	–0.8274	–0.3023	–0.3408	0.1569	2.71	0.8352
**Luminal B**	–3.7511	–0.5011	0.0697	0.0855	0.7793	4.44	1.0467
**Normal breast-like**	–4.4761	–0.5859	–0.0977	–0.1125	0.3917	3.82	0.8434

Dunnett-Tukey-Kramer's test:

gene-expression comparisons				p-value
HER2-E	<	Basal-like		< 0.0001
Luminal A	<	Basal-like		< 0.0001
Luminal A	<	HER2-E		< 0.0001
Luminal B	<	Basal-like		< 0.0001
Luminal B	>	Luminal A		< 0.0001
Normal breast-like	<	Basal-like		< 0.0001

Normal breast-like	>	Luminal A		< 0.0001	
Normal breast-like	<	Luminal B		< 0.01	
Luminal B	=	HER2-E		> 0.10	
Normal breast-like	=	HER2-E		> 0.10	
