# Supplementary material for: Evaluation of Expression and Clinicopathological Relevance of Small Nucleolar RNAs (snoRNAs) in Invasive Breast Cancer
Source: Noncoding RNA. 2025 Oct 31;11(6):76. doi: 10.3390/ncrna11060076 (PMC12642022; doi:10.3390/ncrna11060076)
Supplement: Supplementary file 1 [file ncrna-11-00076-s001.zip › Supplementary file S6.pdf]

Supplementary file S6

Table S5. List of differentially expressed genes and the fold differences between breast cancer-associated plasma (n = 21) and healthy control plasma (n = 20)

**Downregulated expression**					
**Gene**	***P***	**Ratio**	**FD Ct≤40**	**95% CI, low (FD)**	**95% CI, high (FD)**
RNU2-1	0.03715	0.68	–1.48	–2.14	–1.02
SNORA68	*1.00000**	*0.92*	*–1.09*	*–1.61*	*–0.74*
**Upregulated expression**					
**Gene**	***P***	**Ratio/FD Ct≤40**		**95% CI, low**	**95% CI, high**
SCARNA2	*0.08766**	*1.48*		*0.99*	*2.21*
SCARNA3	*0.05459**	*1.31*		*0.97*	*1.77*
SNORD15B	*0.40066**	*1.18*		*0.89*	*1.56*

Notes: Gene expression was normalized to endogenous control gene ACTB. FD = fold difference, CI – confidence interval. Data were not corrected for multiple testing as such data resulted in no significant results. * = non-significant results

Table S6. Results of the receiver operating characteristics analysis for plasma of invasive breast cancer of no special type patients (n = 21) and healthy control samples (n = 20) with respect to gene expression (qPCR, Plasma Experiment)

Variable (gene expression)	*SCARNA2*	*SCARNA3*	*SNORD15B*	*RNU2-1*	*SNORA68*
**Area under the ROC curve (AUC)**	0.657	0.676	0.579	0.690	0.500
**Standard Error**	0.0880	0.0863	0.0923	0.0866	0.0955
**95% Confidence interval [a]**	0.493 to 0.798	0.512 to 0.814	0.414 to 0.731	0.527 to 0.825	0.340 to 0.660
**z statistic**	1.785	2.041	0.851	2.199	0.000
**Significance level *p* (Area=0.5)**	0.0742	0.0413	0.3947	0.0279	1.0000
**Youden index J**	0.3286	0.3595	0.2333	0.4024	0.2024
**95% Confidence interval [a]**	0.1742 to 0.5167	0.1595 to 0.5190	0.1595 to 0.3000	0.1728 to 0.5595	0.1167 to 0.2238
**Associated criterion**	>0.1737	>-0.02582	>0.1166	≤0.1588	>0.2527
**95% Confidence interval**	>0.07359 to >0.4172	>-0.140102187 to >0.179	>0.04601 to >0.3278	≤0.08502 to ≤0.2301	>0.06963 to >0.2722
**Sensitivity (%)**	42.86	80.95	33.33	95.24	4.76
**Specificity (%)**	90.00	55.00	90.00	45.00	75.00
