# Supplementary material for: Evaluation of Expression and Clinicopathological Relevance of Small Nucleolar RNAs (snoRNAs) in Invasive Breast Cancer
Source: Noncoding RNA. 2025 Oct 31;11(6):76. doi: 10.3390/ncrna11060076 (PMC12642022; doi:10.3390/ncrna11060076)
Supplement: Supplementary file 1 [file ncrna-11-00076-s001.zip › Supplementary file S7.pdf]

Supplementary file S7

Table S7

A. Clinicopathological characteristics of patients (Tissue experiment)

**Characteristics**	**Gene expression study (qPCR)**
Number of samples	Screening: eight matched cancer and benign samples. Validation: Cancer samples (n = 22), Control benign samples (n = 20). Nine matched cancer/benign pairs.
Age (years)<br>Median (range) Breast cancer<br>Benign	 69.5 (49-89)<br>69.5 (45-89)
Tumor stage I	13
Tumor stage II	7
Tumor Stage IV	2
Clinical stage I	12
Clinical stage II	6
Clinical stage III	2
Clinical stage IV	2
Histological grade	

G1	4
G2	10
G3	8
Pathological subtypes (cut-off 10%, 15%/20% for Ki-67)	
Ki-67 status (Cut-off 15%)	
Negative	11
Positive	11
Ki-67 status (Cut-off 20%)	
Negative	12
Positive	10
ER status (Cut-off 10%)	
Negative	2
Positive	20
PR status (Cut-off 10%)	
Negative	6
Positive	16
Her2 status	
Negative	21
Positive	1
Multifocal disease	
Negative	19
Positive	3
Lymph node metastasis	
Negative	14
Positive	4
n/a	4
Follow-up weeks (median, range) (n = 21)	308 (34 to 347)

Recurrence/Progression (other than exitus)	3
Exitus	2

Notes: ER, estrogen receptor; Her2, Receptor tyrosine-protein kinase erbB-2; Ki-67: Proliferation marker Ki-67;

PR, progesterone receptor. n/a – not known/available. Tissue samples were collected in the University Hospital Brno (FN Brno).

B. Clinicopathological characteristics of patients (Experiment Plasma)

**Characteristics**	**Gene expression study (qPCR)**
Number of samples	Cancer samples (n = 21), healthy control samples (n = 20).
Age (years)<br>Median (range) Breast cancer patients<br>Healthy controls	 60 (29-74)<br>47.5 (41-62)
Tumor/clinical stage I	14
Tumor/clinical stage II	7
Histological grade	
G1	7
G2	11
G3	3
Pathological subtypes (cut-off 10%, 15%/20% for Ki-67)	
Ki-67 status (Cut-off 15%)	
Negative	7
Positive	14

Ki-67 status (Cut-off 20%)	
Negative	12
Positive	9
ER status (Cut-off 10%)	
Negative	0
Positive	21
PR status (Cut-off 10%)	
Negative	3
Positive	18
Her2 status	
Negative	21
Positive	0
Multifocal disease	
Negative	19
Positive	2
Lymph node metastasis	
Negative	18
Positive	3
Follow-up weeks (median, range) (n = 21)	257 (43-321)
Recurrence/Progression (other than exitus)	0
Exitus	1

Notes: Blood samples were collected at ÚPMD Prague–Podolí (from breast cancer patients) and at the University Hospital in Prague (VFN, Faculty Transfusion Department) from healthy control subjects.
