# Supplementary material for: Evaluation of Expression and Clinicopathological Relevance of Small Nucleolar RNAs (snoRNAs) in Invasive Breast Cancer
Source: Noncoding RNA. 2025 Oct 31;11(6):76. doi: 10.3390/ncrna11060076 (PMC12642022; doi:10.3390/ncrna11060076)
Supplement: Supplementary file 1 [file ncrna-11-00076-s001.zip › Supplementary file S8.pdf]

Supplementary file S8

Table S8. List of the investigated genes and endogenous controls

**Small nucleolar RNA-related genes, additional genes and endogenous control genes analyzed in the screening and in the validation**				
**Gene symbol**	**Gene name**	**Type of RNA**	**Chromosome location of the investigated gene**	**Catalog number/Assay ID (ThermoFisher Scientific)**
SNORD15A	Small Nucleolar RNA, C/D Box 15A	snoRNA	Chr.11: 75400391 - 75400538	Hs04332088_s1
SNORD15B	Small Nucleolar RNA, C/D Box 15B	snoRNA	Chr.11: 75404421 - 75404566	Hs03296837_s1
SNORD16	Small Nucleolar RNA, C/D Box 16	snoRNA	Chr.15: 66502811 - 66502910	Hs06626885_s1
SNORD48	Small Nucleolar RNA, C/D Box 48	snoRNA	Chr.6: 31835263 - 31835326	Hs04931161_g1
SNORD89,RNF149	Small Nucleolar RNA, C/D Box 89	snoRNA	Chr.2: 101272936 - 101273049	Hs03298802_s1
SNORD94	Small Nucleolar RNA, C/D Box 94	snoRNA	Chr.2: 86135870 - 86136006	Hs03309798_s1
SNORD97	Small Nucleolar RNA, C/D Box 97	snoRNA	Chr.11: 10801467 - 10801608	Hs03301409_s1

SNORA68	Small Nucleolar RNA, H/ACA Box 68	snoRNA	Chr.19: 17862588 - 17862720	Hs04416102_s1
SNORA71A	Small Nucleolar RNA, H/ACA Box 71A	snoRNA	Chr.20: 38427306 - 38427443	Hs03309449_s1
SNHG1	Small Nucleolar RNA Host Gene 1	snoRNA host gene, lncRNA	Chr.11: 62851988 - 62855888	Hs00411543_m1
SNHG3	Small Nucleolar RNA Host Gene 3	snoRNA host gene, lncRNA	Chr.1: 28505943 - 28510892	Hs05055352_s1
SNHG5	Small Nucleolar RNA Host Gene 5	snoRNA host gene, lncRNA	Chr.6: 85677007 - 85678733	Hs05037597_s1
SNHG6	Small Nucleolar RNA Host Gene 6	snoRNA host gene, lncRNA	Chr.8: 66921930 - 66925542	Hs00996619_g1
SNHG7,SNORA17B	Small Nucleolar RNA Host Gene 7, Small Nucleolar RNA, H/ACA Box 17B	snoRNA host gene, lncRNA	Chr.9: 136724594 - 136728184	Hs01075370_g1
SNHG8	Small Nucleolar RNA Host Gene 8	snoRNA host gene, lncRNA	Chr.4: 118278762 - 118279823	Hs03461282_g1
SNHG9	Small Nucleolar RNA Host Gene 9	snoRNA host gene, lncRNA	Chr.16: 1964996 - 1965504	Hs03299045_g1
SNHG11	Small Nucleolar RNA Host Gene 11	snoRNA host gene, lncRNA	Chr.20: 38446654 -	Hs00290821_m1

			38450921	
SNHG15	Small Nucleolar RNA Host Gene 15	snoRNA host gene, lncRNA	Chr.7: 44983028 - 44986660	Hs03301064_g1
SNHG16	Small Nucleolar RNA Host Gene 16	snoRNA host gene, lncRNA	Chr.17: 76557764 - 76565348	Hs01598403_g1
SCARNA1	Small Cajal Body-Specific RNA 1	scaRNA	Chr.1: 27834401 - 27834566	Hs03298705_s1
SCARNA2	Small Cajal Body-Specific RNA 2	scaRNA	Chr.1: 109100193 - 109100612	Hs04232660_s1
SCARNA3,RFWD2	Small Cajal Body-Specific RNA 3	scaRNA	Chr.1: 175968397 - 175968540	Hs03298706_s1
SCARNA4	Small Cajal Body-Specific RNA 4	scaRNA	Chr.1: 155925958 - 155926086	Hs03298714_s1
SCARNA5	Small Cajal Body-Specific RNA 5	scaRNA	Chr.2: 233275726 - 233276003	Hs03298717_s1
SCARNA6	Small Cajal Body-Specific RNA 6	scaRNA	Chr.2: 233288676 - 233288941	Hs03298715_s1
SCARNA7	Small Cajal Body-Specific RNA 7	scaRNA	Chr.3: 160514907 - 160515236	Hs03309492_s1
SCARNA8	Small Cajal Body-Specific RNA 8	scaRNA	Chr.9: 19063656 -	Hs03298719_s1

			19063786	
SCARNA9	Small Cajal Body-Specific RNA 9	scaRNA	Chr.11: 93721514 - 93721866	Hs03464471_s1
SCARNA10	Small Cajal Body-Specific RNA 10	scaRNA	Chr.12: 6510222 - 6510551	Hs03309805_s1
SCARNA11	Small Cajal Body-Specific RNA 11	scaRNA	Chr.12: 6581473 - 6581609	Hs04333779_s1
SCARNA12	Small Cajal Body-Specific RNA 12	scaRNA	Chr.12: 6967337 - 6967606	Hs03309494_s1
SCARNA14	Small Cajal Body-Specific RNA 14	scaRNA	Chr.15: 66347206 - 66347342	Hs03309806_s1
SCARNA17	Small Cajal Body-Specific RNA 17	scaRNA	Chr.18: 49814023 - 49814443	Hs03298712_s1
SCARNA18	Small Cajal Body-Specific RNA 18	scaRNA	Chr.5: 83064204 - 83064337	Hs04232679_s1
SCARNA20	Small Cajal Body-Specific RNA 20	scaRNA	Chr.17: 60231516 - 60231645	Hs04333773_s1
SCARNA21	Small Cajal Body-Specific RNA 21	scaRNA	Chr.17: 7906123 - 7906260	Hs03464476_s1
SCARNA22	Small Cajal Body-Specific RNA 22	scaRNA	Chr.4: 1974636 - 1974760	Hs03298713_s1
SCARNA23	Small Cajal Body-	scaRNA	Chr.X:	Hs03309493_s1

	Specific RNA 23		24744441 - 24744570	
SCARNA27	Small Cajal Body-Specific RNA 27	scaRNA	Chr.6: 8086408 - 8086533	Hs06633095_g1
SCARNA28	Small Cajal Body-Specific RNA 28	scaRNA	Chr.7: 98881697 - 98881890	Hs04937981_s1
TERC	Telomerase RNA Component, SCARNA19	telomerase RNA	Chr.3: 169764610 - 169765060	Hs03297287_s1
RNU2-1	RNA, U2 Small Nuclear 1	small nuclear RNA (snRNA)	Chr.17: 43233790 - 43233977	Hs02786874_gH
ACTB	actin beta	endogenous control	Chr.7: 5527148 - 5530601	Hs01060665_g1
GAPDH	glyceraldehyde-3-phosphate dehydrogenase	endogenous control	Chr.12: 6534405 - 6538375	Hs02786624_g1
TBP	TATA-box binding protein	endogenous control	Chr.6: 170554333 - 170572870	Hs00427620_m1
RPLP0	ribosomal protein lateral stalk subunit P0	endogenous control	Chr.12: 120196700 - 120201211	Hs00420895_gH
IPO8	importin 8	endogenous control	Chr.12: 30628981 - 30695995	Hs00914057_m1

18S	Eukaryotic 18S rRNA	endogenous control	Chr. 21	Hs99999901_s1
HPRT1	Hypoxanthine phosphoribosyltransferase 1; HGPRT; HPRT	endogenous control	Chr.X: 134460145 - 134500668	Hs99999909_m1
GUSB	glucuronidase beta; BG; MPS7	endogenous control	Chr.7: 65960684 - 65982314	Hs99999908_m1

Notes: snoRNA = small nucleolar RNA; lncRNA = long non-coding RNA. *SCARNA2*, *SCARNA3*, *SNORD15B*, *SNORD94*, *RNU2-1* (snRNA), *SNHG1*, and *SNORA68* were analyzed also in the validation (tissues). snRNA = small nuclear RNA
